# Coumarin‐Based Antifungal Leads: Design, Synthesis, Mechanistic Insights, and Synergistic Activity

**DOI:** 10.1002/cbdv.71773

**Published:** 2026-09-30

**Authors:** Xiangna Chang, Qiang Du, Xiaoying Ma, Jundi Zhang

**Affiliations:** ^1^ School of Food Science and Engineering Shaanxi University of Science & Technology Xi'an China

**Keywords:** antifungal lead compounds, coumarin derivative, membrane integrity, molecular docking, plant pathogenic fungi, synergistic interactions

## Abstract

To address the challenge of fungicide resistance in plant pathogenic fungi, coumarin derivatives were designed. Two series of coumarin derivatives, including 3‐amide and thienocoumarin ether derivatives, were designed and synthesized through structural modification of the coumarin scaffold to explore their antifungal potential. Their antifungal activities were evaluated using the mycelial growth rate method, with half‐maximal effective concentration (EC_50_) values determined. The effects of compound **IVd** on membrane integrity and morphology were assessed by measuring electrolyte leakage, malondialdehyde (MDA) content, and scanning electron microscopy (SEM). Synergistic effects were quantified using Wadley's method by calculating the synergistic ratio (SR). Molecular docking studies were conducted to investigate interactions with potential targets (CYP51, CYP51B, and SDH). Among the derivatives, **IVd** showed the most potent antifungal activity, with EC_50_ values of 88.5 µg/mL against *Valsa mali*, slightly surpassing azoxystrobin (EC_50_ = 92.8 µg/mL). Treatment with **IVd** caused membrane disruption and hyphal deformation. A 2:1 combination ratio of **IVd** with azoxystrobin or fluconazole demonstrated synergistic effects (SR > 1.5). Molecular docking suggested favorable interactions with the investigated targets, supporting its multitarget activity. Overall, these findings highlight **IVd** as a promising antifungal scaffold for investigation and provide evidence for its potential use in combination strategies against plant pathogenic fungi.

## Graphical abstract

A series of novel coumarin derivatives are designed and synthesized as antifungal leads. The optimized compound **IVd** displays potent activity against plant pathogens, potential multitarget antifungal effects, and synergistic interactions with commercial fungicides, providing a promising strategy for resistance management.

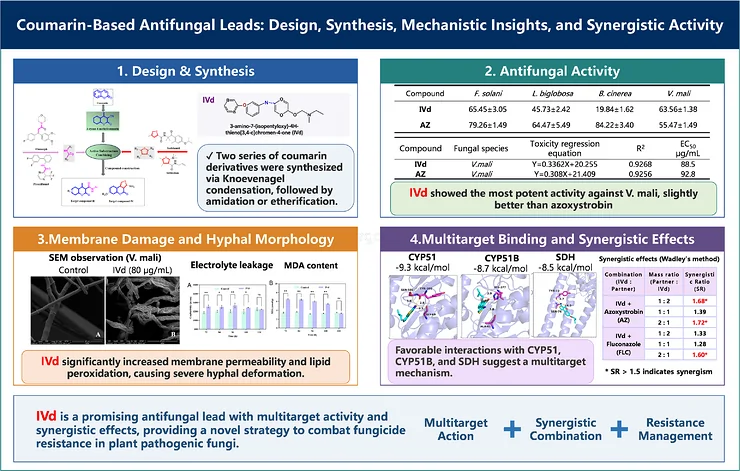

## 1 Introduction

Plant fungal diseases pose a significant threat to global agricultural production, adversely affecting both crop yield and quality [1]. For instance, *Botrytis cinerea* can infect various crops, causing gray mold disease and leading to substantial economic losses [2]. In citrus fruits, green and blue molds caused by *Penicillium* species result in significant postharvest losses [3]. Conventional chemical fungicides face challenges related to toxicity, safety concerns, and residue issues [4, 5]. For example, deoxynivalenol (DON) produced by *Fusarium graminearum* contaminates grains, posing potential risks to human and animal health [6]. Furthermore, the increasing development of fungal resistance to these fungicides, coupled with the declining efficacy of conventional fungicides in crop disease management, has become a prominent issue [7]. These challenges complicate disease control, creating a vicious cycle. Consequently, the development of novel fungicides with diverse chemical scaffolds and improved efficacy has become an important strategy for addressing the challenges associated with fungicide resistance.

To address the challenge of fungal resistance, two strategies have shown promise. The first strategy involves combining fungicides with different modes of action to enhance efficacy through synergistic effects and delay the emergence of resistance. For example, the combination of inhibitors targeting fungal cell membranes and cell walls can improve effectiveness, reduce the dosage requirements of individual fungicides, and consequently lower the risk of resistance development [8]. The second strategy involves the development of compounds with potential multi‐pathway effects, which may provide new opportunities for overcoming resistance. Certain plant‐derived compounds have been reported to affect multiple physiological processes, including fungal cell membrane integrity and intracellular signaling pathways [9]. Such potential multi‐target characteristics may offer valuable insights into the development of novel antifungal agents against plant pathogens.

Compared to traditional synthetic fungicides, natural product derivatives offer advantages such as biodegradability, high safety, and low residue levels, while exhibiting promising antifungal potential [10, 11, 12]. Coumarin, characterized by its benzopyrone structure, is an important secondary metabolite in plants that contributes to defense against pathogen infection and displays a wide range of biological activities, including anticoagulant [13], anti‐HIV [14, 15], antitumor [16, 17, 18], antiviral [19], and notable antimicrobial effects [20, 21]. Moreover, its core scaffold contains multiple modifiable active sites on both the benzene and pyrone rings, allowing for structural optimization [22]. Previous studies have shown that coumarin derivatives, such as pyrrolocoumarins [23] and fluorinated coumarins [24], possess antifungal activities superior to certain commercial fungicides. However, most reported coumarin‐based antifungal agents have mainly focused on conventional substituent modification, while coumarin derivatives incorporating structural features inspired by commercial fungicides and possessing potential synergistic activity remain relatively limited. Therefore, further structural exploration of coumarin scaffolds is still required to develop novel antifungal candidates with improved efficacy and reduced resistance risk. In this study, a 3‐cyano‐4‐methylcoumarin scaffold was synthesized via Knoevenagel condensation, and two novel series of coumarin derivatives were designed by incorporating active structural motifs from commercial fungicides, including flumorph and isofetamid [25, 26, 27, 28]. This strategy may provide new opportunities for developing coumarin‐based antifungal agents, as illustrated in Figure 1. The hydroxyl group was further modified through etherification to regulate the physicochemical properties of the coumarin scaffold, such as polarity and hydrophobicity, and to explore its influence on antifungal activity. These derivatives, comprising coumarin‐3‐carboxamides (series II) and thienocoumarin ethers (series IV), were evaluated for their activity against four plant pathogenic fungi, resulting in the identification of compounds with promising antifungal potential. Their potential for combination therapy was also examined, and preliminary investigations into their possible antifungal modes of action were performed [29]. This work aims to identify novel coumarin‐based antifungal lead compounds, explore their potential synergistic interactions with commercial fungicides, and provide preliminary evidence for their possible antifungal modes of action against plant pathogenic fungi.

**FIGURE 1:**
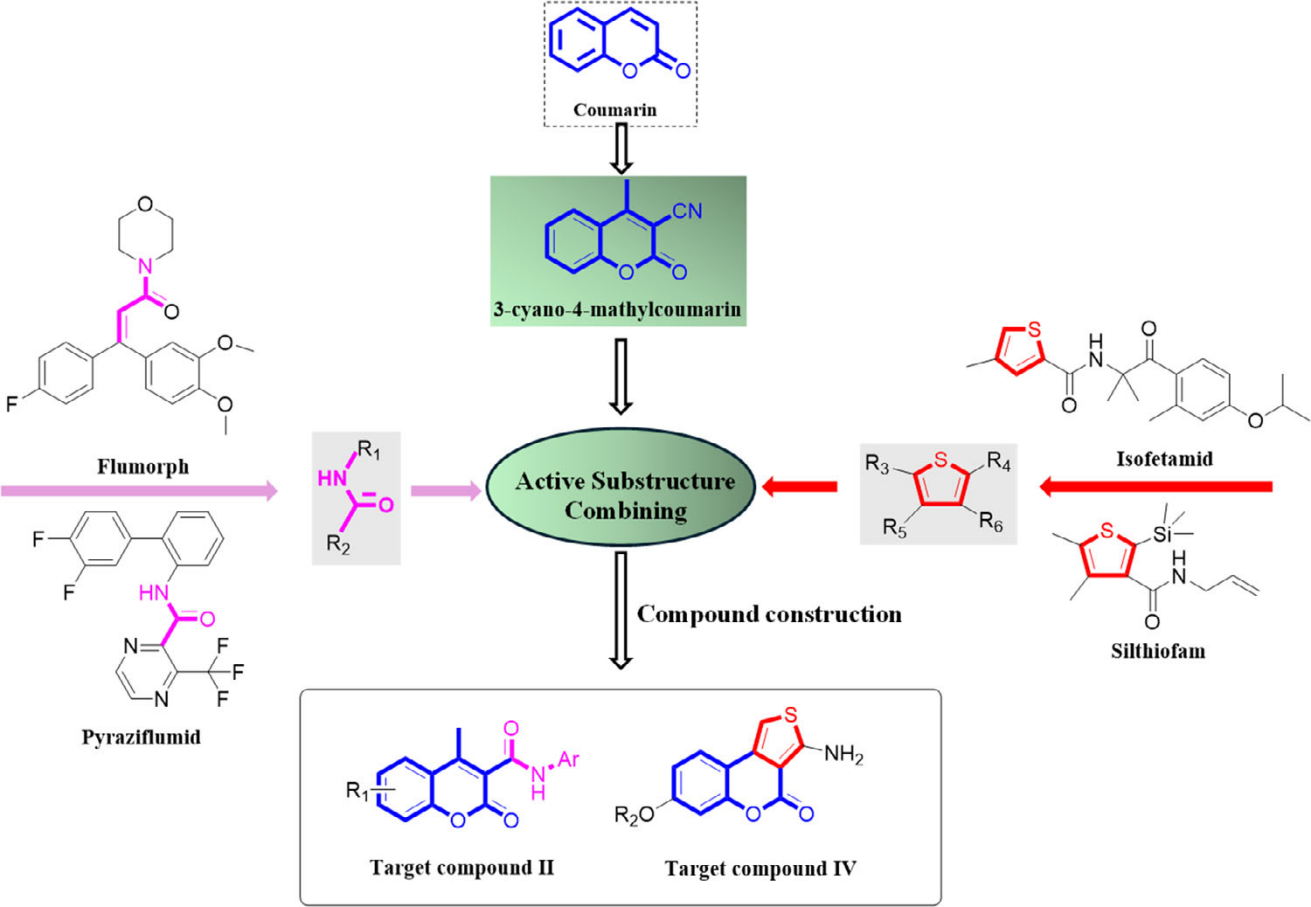
Design strategy for the target coumarin derivatives. The hybrid strategy was based on the 3‐cyano‐4‐methylcoumarin scaffold and active substructures from commercial fungicides (flumorph, pyraziflumid, isofetamid, and silthilfam).

## 2 Results and Discussion

### 2.1 Synthesis

Inspired by the molecular design of commercial amide‐containing fungicides, such as flumorph and isofetamid, a series of coumarin‐3‐carboxamide derivatives (series IIa and IIb) was successfully synthesized via a sequential route involving Knoevenagel condensation, cyano hydrolysis, and amidation. The IIa series, unsubstituted at the 7‐position, was obtained in yields of 54%–72%, whereas the 7‐methoxy‐substituted IIb series gave slightly lower yields of 53%–68%. This discrepancy may be attributed to the electron‐donating effect of the 7‐methoxy group, which reduces the electrophilicity of the carbonyl carbon and thereby decreases the efficiency of the amidation reaction.

### 2.2 Antifungal Activity and Structure‐Activity Relationship (SAR) Analysis

The inhibitory activities of all synthesized compounds against the four plant pathogenic fungi at a concentration of 100 µg/mL are summarized in Table S1. Against *F. solani*, compounds **IVd** (65.45%) and **IIb5** (61.91%) exhibited inhibition rates exceeding 60%, corresponding to 82.6% and 78.1% of the activity of the positive control azoxystrobin (79.26%), respectively. Notably, both active compounds incorporate heterocyclic motifs (either an annulated aminothiophene or a thiazole substituent), suggesting that the introduction of heterocycles may enhance antimicrobial potency. For *L. biglobosa*, compound **IVd** demonstrated the highest activity (45.73%), while the majority of compounds showed limited inhibition (< 17%). This indicates a generally lower sensitivity of this fungus to the core coumarin scaffold; however, the annulated aminothiophene structure appears to confer a relative advantage. Against *V. mali*, the series IV compounds demonstrated significantly higher activity compared to other structural classes. Notably, compound **IVd** exhibited superior inhibitory activity (63.56%) against *V. mali* compared to the positive control azoxystrobin (55.47%), indicating a pronounced advantage of the aminothienyl‐annulated scaffold against this pathogen. This enhanced potency may be attributed to the optimized spatial conformation and electronic distribution of the aminothiophene moiety, potentially facilitating stronger interactions with the target protein(s).

Further SAR analysis indicated that structural modification of the coumarin scaffold influenced antifungal activity [30]. Compared with coumarin‐3‐carboxamide derivatives (series II), thienocoumarin ether derivatives (series IV) showed improved activity, suggesting that heterocyclic fusion and sulfur incorporation may contribute to enhanced antifungal potency by altering molecular properties [31]. The aminothiophene moiety and hydroxyl etherification in IVd may improve molecular polarizability and the balance between polarity and lipophilicity, while the electron‐donating alkoxy group at the 7‐position may favor ligand–target interactions [32, 33]. These results highlight the importance of heterocyclic incorporation and substituent modification in optimizing coumarin‐based antifungal agents [34].

Comprehensive analysis revealed that **IVd**, an aminothienocoumarin ether derivative, showed the most promising broad‐spectrum antifungal activity, demonstrating notable efficacy against three key pathogens: *F. solani*, *L. biglobosa*, and *V. mali*. Furthermore, its half‐maximal effective concentration (EC_50_) against *V. mali* was determined to be 88.5 µg/mL, which is lower than that of azoxystrobin (AZ; EC_50_ = 92.8 µg/mL) (Table S2), indicating greater potency. Based on its relatively favorable antifungal profile and distinct structural features, compound **IVd** was selected for subsequent studies on synergistic effects and preliminary mechanism exploration.

### 2.3 Synergistic Antifungal Effects of **IVd** With Positive Control Agents

Previous studies have shown that drug combinations can enhance efficacy and reduce individual dosages through synergistic interactions, thereby facilitating the restoration of drug susceptibility in resistant strains [35]. This study evaluated the synergistic effects of **IVd** in combination with azoxystrobin (AZ) and fluconazole (FLC). As summarized in Table S3, the EC_50_ values for the AZ:**IVd** combinations at 1:1 and 2:1 mass ratios were significantly lower compared to the individual agents alone. Furthermore, all tested combinations of FLC with **IVd** exhibited superior activity compared to FLC alone. Analysis of the synergistic ratio (SR) revealed that the AZ:**IVd** (1:2, 1:1) and FLC:**IVd** (1:2, 1:1) combinations yielded SR values ranging from 0.92 to 1.27, indicative of an additive effect. In contrast, the 2:1 ratio combinations of both AZ: **IVd** and FLC: **IVd** resulted in SR values exceeding 1.5, suggesting significant synergism. Consistently, the EC_50_ values of the synergistic combinations were markedly reduced compared with those of the individual compounds. For example, the AZ:**IVd** (1:1) and AZ:**IVd** (2:1) combinations exhibited EC_50_ values of 76.42 and 53.17 µg/mL, respectively, which were lower than those of AZ (92.83 µg/mL) and **IVd** (88.47 µg/mL) alone. Particularly, the AZ:**IVd** (2:1) combination showed approximately 1.7‐fold improvement in antifungal activity compared with AZ alone. Similarly, FLC:**IVd** (1:1) and FLC:**IVd** (2:1) combinations displayed EC_50_ values of 63.48 and 49.64 µg/mL, respectively, outperforming FLC (73.84 µg/mL) and **IVd** (88.47 µg/mL) alone. Notably, the aminothienocoumarin ether scaffold of **IVd** is structurally distinct from conventional antifungal classes such as azoles [36] and diazines [37]. This structural novelty suggests that its use in combination with existing fungicides could facilitate dose reduction, thereby potentially reducing resistance pressure associated with repeated application of single fungicides. Therefore, the observed synergistic interactions of **IVd** with commercial fungicides provide preliminary support for its further evaluation in antifungal combination systems against plant pathogenic fungi. The underlying mechanism of this synergism warrants further investigation.

### 2.4 Effects of **IVd** on Membrane Integrity and Lipid Peroxidation in *V. mali* Hyphae

As shown in Figure 2A, the extracellular conductivity of *V. mali* hyphae treated with 80 µg/mL **IVd** exhibited significant changes during the 72–120 h cultivation period. From 72 to 108 h, the conductivity in the **IVd**‐treated group remained consistently higher than that in the blank control group (*p <* 0.05), indicating that **IVd** disrupted membrane permeability and caused intracellular ion leakage [29, 38]. Notably, conductivity peaked at 96 h and subsequently declined, possibly reflecting a dynamic balance between membrane damage and repair processes. Malondialdehyde (MDA), a hallmark of lipid peroxidation [46], was used as an indicator of oxidative damage. As shown in Figure 2B, MDA levels in the **IVd**‐treated group peaked at 72 h (6.51 nmol/g), significantly higher than those in the control group (3.48 nmol/g). From 72 to 108 h, MDA levels in the treated group remained significantly elevated (*p <* 0.05), indicating that **IVd** induced lipid peroxidation in *V. mali*. Such peroxidation markedly disrupts membrane structure and function, thereby causing further membrane damage [39, 40]. In summary, **IVd** likely induces membrane damage through a synergistic mechanism involving increased membrane permeability and lipid peroxidation, consistent with the report by Wang et al. [41] that the coumarin derivative esculetin caused membrane damage in *Phytophthora capsici*.

**FIGURE 2:**
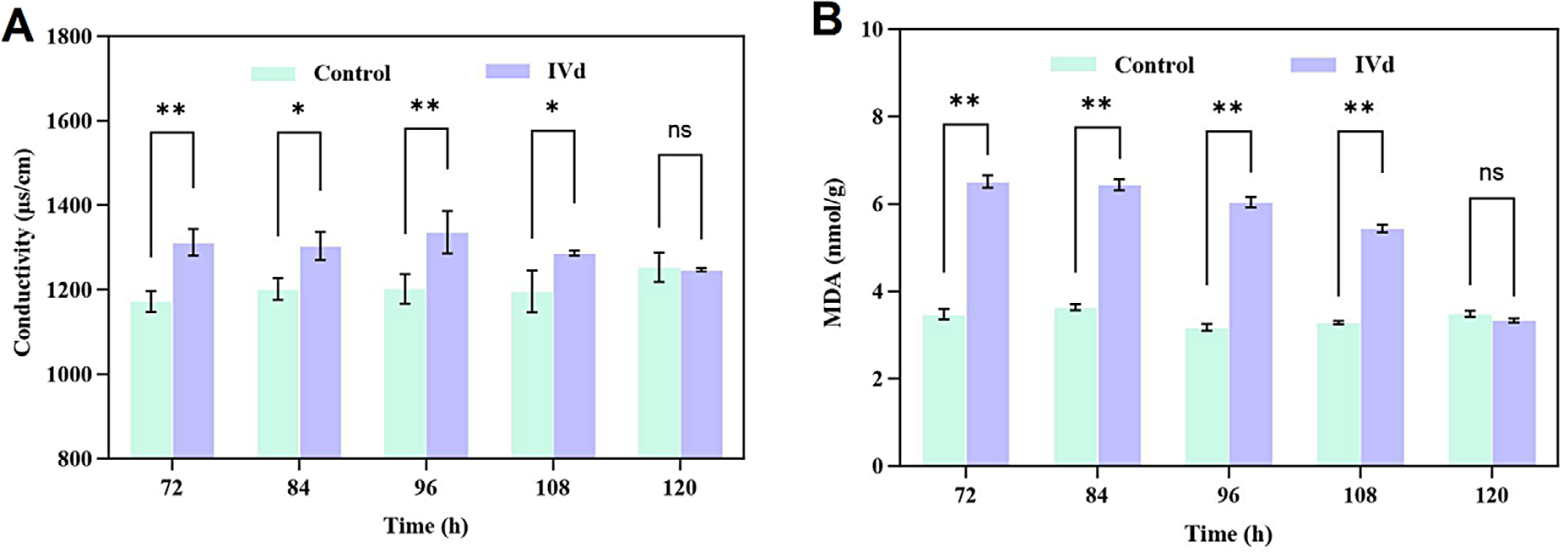
Effects of **IVd** on membrane integrity and lipid peroxidation in *V. mali*. (A) Extracellular conductivity and (B) malondialdehyde (MDA) content of mycelia cultured in PDB medium with or without **IVd** (80 µg/mL) for 72–120 h. Data were collected at 72, 84, 96, 108, and 120 h and are expressed as mean ± SD (*n* = 3). Statistical significance was assessed using one‐way ANOVA followed by the LSD post hoc test. *p < 0.05, **p < 0.01 versus the control group at the corresponding time point; ns, not significant.

### 2.5 Morphological Alterations of *V. mali* Hyphae Induced by Compound **IVd**

Scanning electron microscopy (SEM) was employed to observe the morphological effects of **IVd** on *V. mali* hyphae (Figure 3). Hyphae in the control group exhibited intact and turgid structures with smooth surfaces, indicative of healthy and normal growth (Figure 3A). In contrast, hyphae in the **IVd**‐treated group (Figure 3B) showed severe deformation, with surface damage, twisting, and shrinkage. These morphological abnormalities suggest a loss of cell membrane integrity [42], leading to the leakage of ions and intracellular contents, disruption of osmotic balance [43], and subsequent cellular contraction and deformation [44]. These observations demonstrate the damaging effect of **IVd** on the cell membrane of *V. mali*, consistent with the findings described above.

**FIGURE 3:**
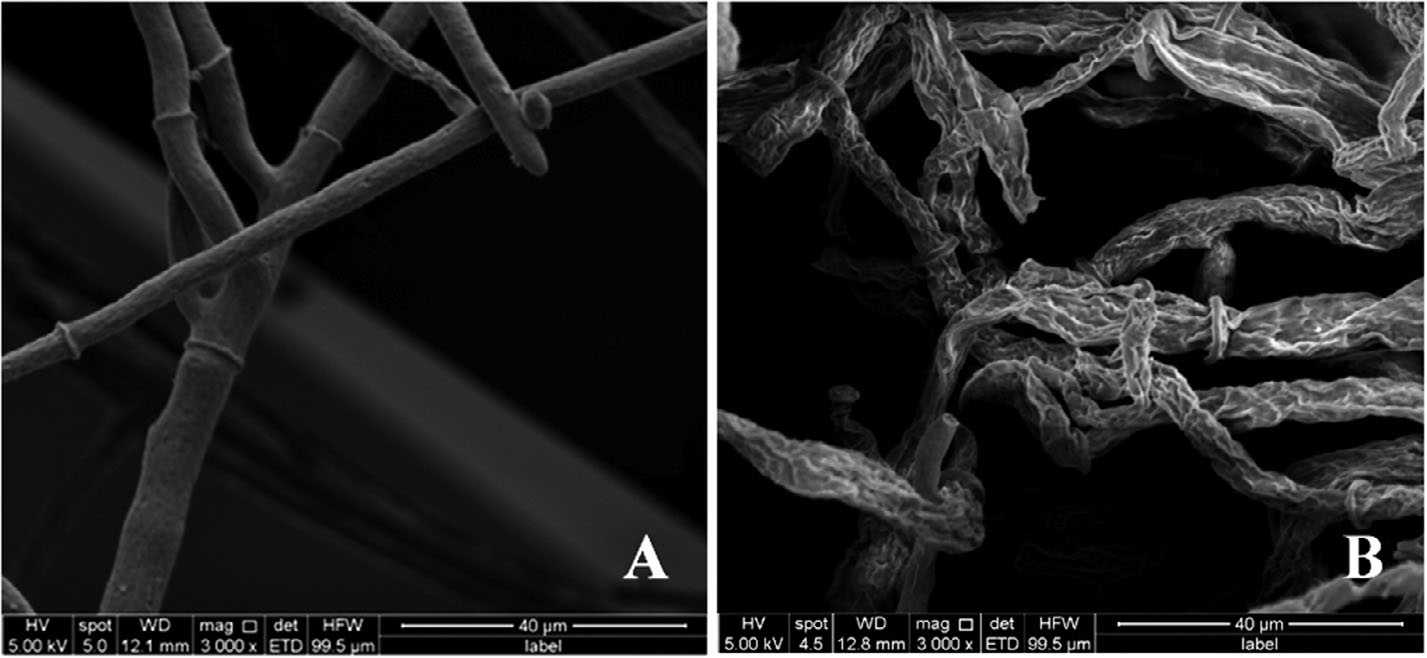
SEM images of *V. mali* mycelia after **IVd** treatment. Mycelia were collected from PDA plates after treatment with (B) 80 µg/mL **IVd** or (A) no drug (control) for 3 days. Samples were processed as described in the Methods. Scale bar, 40 µm (original magnification ×3000).

### 2.6 Molecular Docking Analysis of **IVd** With Potential Target Proteins

Given previous findings that **IVd**0 disrupts membrane integrity and induces lipid peroxidation in *V. mali*, we sought to further investigate its molecular mechanism by selecting three potential target proteins for molecular docking based on literature reports: CYP51B (PDB: 6CR2), CYP51 (PDB: 5FSA), and SDH (PDB: 2FBW) [45, 46]. Docking results revealed that **IVd** could form hydrogen bond interactions with all three targets. The binding energies for CYP51B and CYP51 were −8.4 and −8.0 kcal/mol, respectively, significantly lower than that for SDH (−6.8 kcal/mol) (Table S4), suggesting stable binding affinity for the CYP51 family of proteins [45]. Detailed interaction patterns are depicted in Figure 4. Interaction analysis indicated that **IVd** formed the most stable complex with CYP51B, engaging in three hydrogen bonds with residues TYR500, GLY69, and SER501 within the active site pocket, with bond lengths ranging from 2.8 to 3.6 Å (Figure 4A,a). With CYP51, **IVd** formed two hydrogen bonds with ALA61 and HIS377; however, the longer bond distances (3.8–3.9 Å) were consistent with its weaker binding energy compared with CYP51B (Figure 4B,b). The interaction with SDH was the weakest, characterized by two hydrogen bonds with TYR73 and SER76, with bond lengths of 3.7–4.0 Å (Figure 4C,c). Notably, the aminothiophene moiety of **IVd** was involved in forming key hydrogen bonds with all three targets, highlighting its critical role in ligand–target recognition and corroborating our earlier hypothesis that this structural feature enhances complementary binding to the target protein.

**FIGURE 4:**
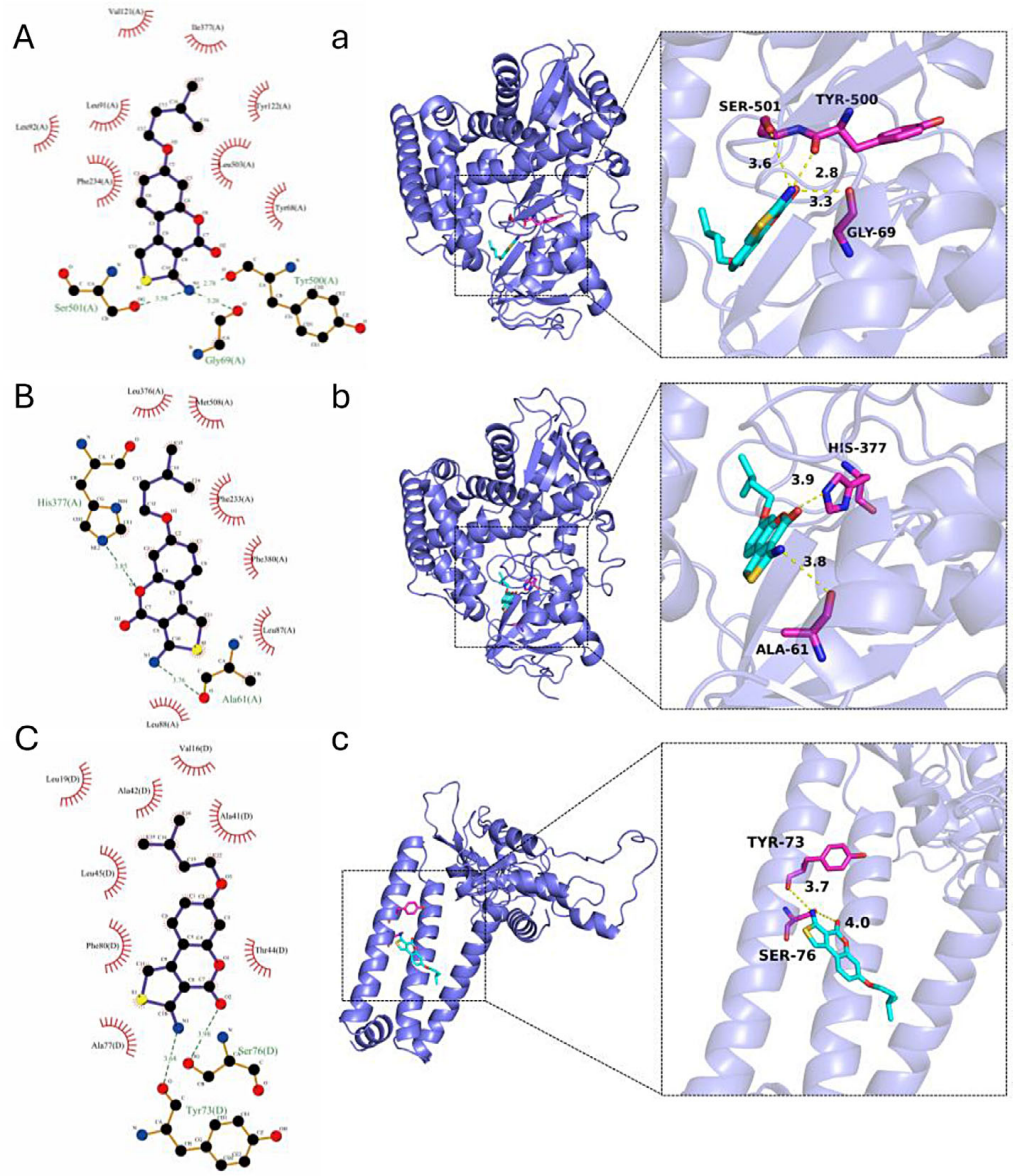
Molecular docking of compound **IVd** with potential target proteins. Two‐dimensional (left) and three‐dimensional (right) interaction diagrams of **IVd** with (A, a) CYP51B (binding energy: −8.4 kcal/mol), (B, b) CYP51 (−8.0 kcal/mol), and (C, c) SDH (−6.8 kcal/mol). Hydrogen bonds, ionic interactions, and hydrophobic interactions are shown as yellow, magenta, and green dashed lines, respectively.

The docking results provide supportive computational evidence that **IVd** may interact with multiple fungal targets. CYP51 and CYP51B are key enzymes in ergosterol biosynthesis, and their inhibition directly compromises membrane integrity [46]. Conversely, SDH, a core subunit of mitochondrial complex II (succinate dehydrogenase), is essential for energy metabolism, and its inhibition disrupts this process [45]. The binding of **IVd** to these three targets suggests that its antifungal efficacy may be mediated through a synergistic mechanism. Specifically, potent inhibition of the CYP51 family impedes ergosterol biosynthesis, leading to structural defects and increased cell membrane permeability, which provides a direct explanation for the experimentally observed increase in extracellular conductivity. Concurrently, inhibition of SDH likely disrupts mitochondrial function, resulting in an accumulation of reactive oxygen species (ROS) [47], which subsequently induces lipid peroxidation and elevates malondialdehyde (MDA) levels. Membrane instability caused by CYP51 inhibition may further potentiate the damaging effects of reactive oxygen species (ROS). Therefore, **IVd** appears to disrupt membrane function synergistically via both direct (potential interference with membrane synthesis) and indirect (possible disruption of energy metabolism → oxidative stress) pathways, a model consistent with our experimental findings. Notably, **IVd** exhibits a potential polypharmacological profile. Compared with conventional single‐target agents, multitarget inhibitors may provide a higher resistance barrier [48]. In this study, **IVd** effectively engaged key targets in distinct pathways—namely, membrane synthesis (CYP51/CYP51B) and energy metabolism (SDH). This suggests a potentially higher resistance barrier through effects on multiple pathways, although further validation is required. Overall, the results suggest that **IVd** may possess potential multitarget antifungal activity and support its further investigation as a coumarin‐based antifungal lead compound.

## 3 Conclusions

In this study, two novel series of coumarin derivatives were designed, synthesized, and systematically evaluated for their antifungal activities against plant pathogenic fungi. Among them, the thienocoumarin ether derivative **IVd** exhibited the most potent broad‐spectrum antifungal activity. Notably, its activity against *V. mali* (EC_50_ = 88.5 µg/mL) was superior to that of the commercial fungicide azoxystrobin (EC_50_ = 92.8 µg/mL). Physiological and morphological investigations suggested that **IVd** may induce membrane damage, oxidative stress, and hyphal deformation in fungal cells. Molecular docking results indicated that **IVd** can effectively bind to key targets in the fungal ergosterol biosynthesis (CYP51/CYP51B) and energy metabolism (SDH) pathways, suggesting a multitarget mechanism of action. Combination assays further confirmed significant synergistic effects (SR > 1.5) when **IVd** was combined with azoxystrobin or fluconazole at a fixed ratio (2:1). This study suggests that **IVd** is not only a promising antifungal lead compound with a potential multitarget profile but also represents a structurally distinct thienocoumarin ether scaffold compared with previously reported coumarin‐based antifungal derivatives. The observed synergistic interactions with commercial fungicides further highlight its potential application as a combination antifungal candidate to improve efficacy and potentially reduce resistance development. Therefore, **IVd** represents a valuable template molecule for the development of novel antifungal agents. Further studies, including target validation, resistance evolution assessment, field efficacy evaluation, and structural optimization, are required to further clarify the antifungal mechanism and explore the practical application potential of this scaffold.

## 4 Experimental Section

### 4.1 Chemicals

Triethylamine, potassium carbonate, ammonium acetate, ethyl cyanoacetate, sodium hydroxide, thionyl chloride, various substituted o‐hydroxyacetophenones, sublimed sulfur, piperidine, trifluoromethanesulfonic anhydride, isobutyl chloroformate, 3‐bromo‐2‐methylpropene, and 3‐(bromomethyl)pentane (reagent grade) were purchased from Beijing Innochem Science & Technology Co., Ltd. Solvents, including tetrahydrofuran (THF), N,N‐dimethylformamide (DMF), dichloromethane (DCM), and ethyl acetate, were obtained from Energy Chemical Co., Ltd. Azoxystrobin (AZ) and fluconazole (FLC) (reagent grade), used as positive controls, were supplied by Shanghai Aladdin Biochemical Technology Co., Ltd. The malondialdehyde (MDA) assay kit was purchased from Nanjing Jiancheng Bioengineering Institute.

### 4.2 Synthesis Procedures

The synthetic routes of all target compounds are shown in Scheme 1, and detailed procedures are as follows:

**SCHEME 1:**
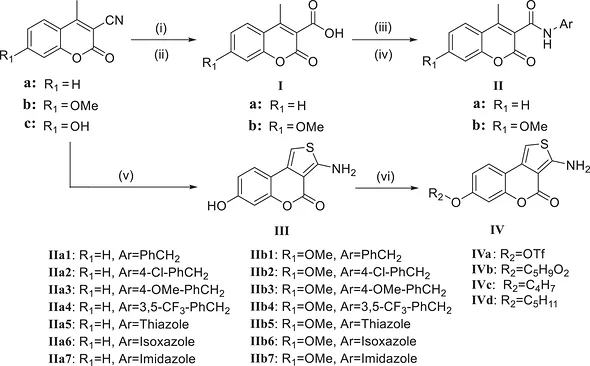
Synthetic route for target compounds **II** and **IV**. Reagents and conditions: (I) conc. H_2_SO_4_, 95°C, 1 h; (II) 5% NaOH, reflux 2 h; (III) SOCl_2_, THF, reflux 1.5 h; (IV) Ar‐NH_2_, 0°C to rt, 3 h; (V) sulfur powder (S_8_), piperidine, DMF, reflux, 4 h; (VI) K_2_CO_3_, acetone, reflux, 6–8 h.

Compounds **a–c** were synthesized according to a reported procedure [49]. Substituted o‐hydroxyacetophenone (10 mmol), ammonium acetate (25 mmol), and ethyl cyanoacetate (15 mmol) were mixed, stirred at room temperature for 3 min, and heated at 130°C–150°C for 2.5 h (monitored by TLC). After cooling to room temperature, 95% ethanol (100 mL) was added, and the mixture was stirred for 6 h. The precipitate was collected by filtration, washed with ethanol, and dried to afford the product.

### 4.3 Synthesis of Intermediates **Ia** and **Ib**

Compounds a or b (5.4 mmol) were placed in a reaction flask, and concentrated H_2_SO_4_ (5 mL) was added. The mixture was heated at 95°C for 1 h. After cooling to room temperature, the mixture was poured into ice–water, stirred for 20 min, and filtered. The resulting solid was recrystallized from ethanol and dried to yield coumarin‐3‐carboxamide. Coumarin‐3‐carboxamide (3.13 mmol) was then placed in a 100 mL round‐bottom flask and treated with 5% NaOH solution (20 mL). The mixture was refluxed for 2 h, cooled to room temperature, poured into ice–water, and acidified with 10% HCl. After stirring at room temperature for 20 min, the solid was filtered, recrystallized from 95% ethanol, and dried to afford intermediates Ia and Ib.

### 4.4 Synthesis of Compounds **IIa1–IIb7**

Compounds Ia or Ib (0.6 mmol) were mixed with thionyl chloride (SOCl_2_, 1.2 mL). After refluxing for 1.5 h, the excess SOCl_2_ was removed by reduced‐pressure distillation. The residue was dissolved in anhydrous tetrahydrofuran (THF, 4 mL) added dropwise to a cooled (0°C) solution of an aromatic amine (0.5 mmol in 2 mL anhydrous THF). Subsequently, triethylamine (0.6 mmol) was introduced, and the mixture was stirred at room temperature for 3 h. Water (15 mL) was then added, and the products were extracted with dichloromethane (3 × 10 mL). The combined organic phases were dried over anhydrous sodium sulfate (Na_2_SO_4_) and concentrated under reduced pressure. Purification by column chromatography (eluent: dichloromethane/ethyl acetate, 20:1 v/v) yielded the target compounds **IIa1–IIb7**.

### 4.5 Synthesis of Compound **III**

A mixture of compound c (10 mmol) and sulfur powder (10 mmol) in DMF (20 mL) was prepared. Piperidine (0.2 mL) was added dropwise, and the reaction mixture was refluxed for 4 h. The formation of compound **III** is proposed to proceed through a Gewald‐type sulfur‐mediated cyclization process. Piperidine promotes deprotonation of the methyl group, followed by sulfur incorporation and intramolecular cyclization with the cyano group to construct the aminothiophene‐fused coumarin scaffold [50]. After cooling to room temperature, the mixture was poured into ice water, stirred for 20 min, and filtered. The solid was dried to give compound III as a dark purple solid.

### 4.6 Synthesis of Compounds **IVa–IVd**

Compound **III** (1.0 mmol) was dissolved in acetone (10 mL), and K_2_CO_3_ (2.0 mmol) was added. The mixture was stirred until the solids dissolved completely, followed by dropwise addition of the corresponding halide (1.5 mmol). The reaction mixture was heated under reflux for 6–8 h, monitored by TLC. After cooling, the mixture was concentrated under reduced pressure, treated with water, and extracted with dichloromethane. The organic phase was dried over anhydrous Na_2_SO_4_, concentrated, and purified by column chromatography (eluent: dichloromethane/ethyl acetate, 15:1 v/v) to afford compounds **IVa–IVd**.

All synthesized compounds were characterized and confirmed by ^1^H NMR, ^1^ ^3^C NMR (Bruker 600 MHz NMR spectrometer), and HRMS (Bruker solanX70 FT‐MS/Agilent 6540 Q‐TOF). FT‐IR spectra were recorded using a Bruker Vertex 70v vacuum infrared spectrometer, and melting points were determined using a precision micro melting point apparatus (X‐5; maximum temperature: 320°C).

#### 4.6.1 *N*‐Benzyl‐4‐Methyl‐2‐Oxo‐2*H*‐Chromene‐3‐Carboxamide (**IIa1**)

White solid powder, 72% yield; melting point: 146.6°C–149.6°C; **FT‐IR (KBr, cm^−1^)**: 3359 cm^−^^1^ (N─H), 3074, 3041 cm^−^^1^ (C─H), 2924 cm^−^^1^ (C─H), 1695 cm^−^^1^ (C═O), 1658 cm^−^^1^ (C═O), 1609 cm^−^^1^ (C═C), 1527 cm^−^^1^ (amide II), 1252 cm^−^^1^ (C─O─C), 1186, 1143 cm^−^^1^ (C─O), 968 cm^−^^1^ (lactone), 759 cm^−^^1^ (C─H), 726, 693 cm^−^^1^ (C─H), 658 cm^−^^1^ (aromatic) (Figure S1); ^1^H NMR (600 MHz, CDCl_3_) δ 7.79 (d, *J* = 8.2 Hz, 1H, H‐5), 7.72 (s, 1H, −NH), 7.60 (t, *J* = 7.7 Hz, 1H, H‐6), 7.39 (d, *J* = 7.1 Hz, 2H, H‐2′, H‐6′), 7.35 (t, *J* = 7.8 Hz, 4H, H‐7, H‐8, H‐3′, H‐5′, overlapping), 7.29 (d, *J* = 7.3 Hz, 1H, H‐4′), 4.65 (d, *J* = 5.8 Hz, 2H, −CH_2_NH−), 2.77 (s, 3H, 4‐CH_3_) ppm (Figure S2); ^13^C NMR (151 MHz, CDCl_3_) δ 164.05, 160.15, 155.31, 152.58, 138.00, 133.03, 128.79, 127.75, 127.52, 125.91, 124.89, 120.52, 120.22, 117.01, 43.90, 16.71 ppm. HR‐ESI‐MS (m/z): calcd for C_18_H_15_NO_3_ [M + H]^+^: 294.1125, found 294.1114.

#### 4.6.2 *N*‐(4‐Chlorobenzyl)‐4‐Methyl‐2‐Oxo‐2*H*‐Chromene‐3‐Carboxamide (**IIa2**)

White solid powder, 69% yield; melting point: 170.9°C–171.4°C; **FT‐IR (KBr, cm^−^^1^)**: 3361 cm^−^^1^ (N─H), 3047 cm^−^^1^ (C─H), 2913 cm^−^^1^ (C─H), 1699 cm^−^^1^ (C═O), 1609 cm^−^^1^ (C═C), 1527 cm^−^^1^ (amide II), 1492 cm^−^^1^ (aromatic), 1254 cm^−^^1^ (C─O─C), 1179 cm^−^^1^ (C─O), 1032 cm^−^^1^ (C─O), 968 cm^−^^1^ (lactone), 812 cm^−^^1^ (C─H), 757 cm^−^^1^ (C─H), 627 cm^−^^1^ (C─Cl) (Figure S3); ^1^H NMR (600 MHz, CDCl_3_) δ 7.85 (s, 1H, −NH), 7.80 (dd, *J* = 8.0, 1.6 Hz, 1H, H‐5), 7.63 – 7.59 (m, 1H, H‐6), 7.40 – 7.29 (m, 6H, H‐7, H‐8, H‐2′, H‐3′, H‐5′, H‐6′), 4.61 (d, *J* = 5.8 Hz, 2H, −CH_2_NH−), 2.77 (s, 3H, 4‐CH_3_) ppm (Figure S4); ^13^C NMR (101 MHz, CDCl_3_) δ 164.14, 160.43, 156.31, 152.61, 136.68, 133.32, 133.27, 129.12, 128.95, 126.05, 125.03, 120.33, 119.92, 117.10, 43.26, 16.87 ppm. HR‐ESI‐MS (m/z): calcd for C_18_H_14_ClNO_3_ [M + H]^+^: 328.0735, found 328.0740 (Figures S3–S4).

#### 4.6.3 *N*‐(4‐Methoxybenzyl)‐4‐Methyl‐2‐Oxo‐2*H*‐Chromene‐3‐Carboxamide (**IIa3**)

White solid powder, 74% yield; melting point: 185.8°C–187.3°C; **FT‐IR (KBr, cm^−^^1^)**: 3349, 3252 cm^−^^1^ (N─H), 3061 cm^−^^1^ (C─H), 2994, 2946 cm^−^^1^ (C─H), 2844 cm^−^^1^ (OCH_3_), 1695 cm^−^^1^ (C═O), 1638 cm^−^^1^ (C═O), 1611 cm^−^^1^ (C═C), 1519 cm^−^^1^ (aromatic), 1246 cm^−^^1^ (C─O─C), 1180 cm^−^^1^ (C─O), 1032 cm^−^^1^ (C─O), 968 cm^−^^1^ (lactone), 812 cm^−^^1^ (C─H), 759 cm^−^^1^ (C─H) (Figure S5); ^1^H NMR (600 MHz, CDCl_3_) δ 7.79 (d, *J* = 8.2 Hz, 1H, H‐5), 7.60 (t, *J* = 7.0 Hz, 1H, H‐6), 7.60 (s, 1H, −NH, overlapping with H‐6), 7.38–7.34 (m, 2H, H‐7, H‐8), 7.32 (d, *J* = 8.7 Hz, 2H, H‐2′, H‐6′), 6.89 (d, *J* = 8.7 Hz, 2H, H‐3′, H‐5′), 4.58 (d, *J* = 5.6 Hz, 2H, −CH_2_NH−), 3.80 (s, 3H, 4′‐OCH_3_), 2.77 (s, 3H, 4‐CH_3_) ppm (Figure S6); ^13^C NMR (101 MHz, CDCl_3_) δ 163.93, 160.25, 159.13, 155.55, 152.65, 133.09, 130.11, 129.21, 125.96, 124.93, 120.48, 120.33, 117.09, 114.24, 55.44, 43.50, 16.80 ppm. HR‐ESI‐MS (m/z): calcd for C_19_H_17_NO_4_ [M + H]^+^: 346.1050, found 346.1057.

#### 4.6.4 *N*‐(3,5‐Bis(Trifluoromethyl)Benzyl)‐4‐Methyl‐2‐Oxo‐2*H*‐Chromene‐3‐Carboxamide (**IIa4**)

White solid powder, 72% yield; melting point: 225.3°C–227.9°C; **FT‐IR (KBr, cm^−^^1^)**: 3320 cm^−^^1^ (N─H), 3078 cm^−^^1^ (C─H), 2934 cm^−^^1^ (C─H), 1712 cm^−^^1^ (C═O), 1661 cm^−^^1^ (C═O), 1615 cm^−^^1^ (C═C), 1379, 1357 cm^−^^1^ (CF_3_), 1281, 1168, 1120 cm^−^^1^ (C─F), 977 cm^−^^1^ (lactone), 753 cm^−^^1^ (C─H), 679 cm^−^^1^ (aromatic) (Figure S7); ^1^H NMR (600 MHz, CDCl_3_) δ 8.24 (s, 1H, −NH), 7.86 (s, 2H, H‐2′, H‐6′), 7.84 (dd, *J* = 8.1, 1.5 Hz, 1H, H‐5), 7.79 (s, 1H, H‐4′), 7.64 (ddd, *J* = 8.5, 7.3, 1.5 Hz, 1H, H‐6), 7.41 – 7.37 (m, 2H, H‐7, H‐8), 4.77 (d, *J* = 6.0 Hz, 2H, −CH_2_NH−), 2.82 (s, 3H, 4‐CH_3_) ppm (Figure S8); ^13^C NMR (151 MHz, CDCl_3_) δ 164.42, 160.65, 157.63, 152.51, 140.96, 133.47, 132.07, 131.84, 127.55, 126.11, 125.05, 121.38, 120.26, 118.84, 117.05, 42.89, 16.80 ppm. HR‐ESI‐MS (m/z): calcd for C_20_H_13_F_6_NO_3_ [M + H]^+^: 430.0872, found 430.0879.

#### 4.6.5 4‐Methyl‐2‐Oxo‐*N*‐(Thiazol‐2‐yl)‐2*H*‐Chromene‐3‐Carboxamide (**IIa5**)

Light yellow solid powder, 64% yield; melting point: 242.8°C–246.4°C; **FT‐IR (KBr, cm^−^^1^)**: 3228, 3201 cm^−^^1^ (N─H), 1661 cm^−^^1^ (C═O), 1605 cm^−^^1^ (C═N/C═C), 1529, 1484 cm^−^^1^ (aromatic), 1451 cm^−^^1^ (C═C), 1194, 1163 cm^−^^1^ (C─N/C─O), 972 cm^−^^1^ (lactone), 761, 734 cm^−^^1^ (C─H) (Figure S9); ^1^H NMR (400 MHz, CDCl_3_) δ 12.03 (s, 1H, −NH), 7.90 (d, *J* = 6.9 Hz, 1H, H‐5), 7.68 (t, *J* = 8.5 Hz, 1H, H‐6), 7.41 (m, 3H, H‐7, H‐8, thiazole‐H‐4′, overlapping), 6.99 (d, *J* = 3.6 Hz, 1H, thiazole‐H‐5′), 2.95 (s, 3H, 4‐CH_3_) ppm (Figure S10); ^13^C NMR (151 MHz, CDCl_3_) δ 164.70, 161.83, 160.19, 153.74, 152.09, 133.50, 129.01, 125.92, 124.39, 120.19, 117.67, 114.10, 112.57, 16.99 ppm. HR‐ESI‐MS (m/z): calcd for C_14_H_10_N_2_O_3_S [M + H]^+^: 287.0485, found 287.0490.

#### 4.6.6 *N*‐(Isoxazol‐3‐yl)‐4‐Methyl‐2‐Oxo‐2*H*‐Chromene‐3‐Carboxamide (**IIa 6**)

White solid powder, 61% yield; melting point: 217.6°C–225.9°C; **FT‐IR (KBr, cm^−^^1^)**: 3359, 3197 cm^−^^1^ (N─H), 1708 cm^−^^1^ (C═O), 1589 cm^−^^1^ (C═N), 1535 cm^−^^1^ (C═C), 1034 cm^−^^1^ (N─O), 989, 958 cm^−^^1^ (heterocycle), 761 cm^−^^1^ (C─H arom.), 790, 710, 664 cm^−^^1^ (ring deformation) (Figure S11); ^1^H NMR (400 MHz, CDCl_3_) δ 10.58 (s, 1H, −NH), 8.30 (s, 1H, isoxazole‐H‐5′), 7.86 (d, *J* = 7.8 Hz, 1H, H‐5), 7.65 (t, *J* = 7.8 Hz, 1H, H‐6), 7.38 (m, 2H, H‐7, H‐8), 7.11 (s, 1H, isoxazole‐H‐4′), 2.88 (s, 3H, 4‐CH_3_) ppm (Figure S12); ^13^C NMR (101 MHz, CDCl_3_) δ 161.88, 160.70, 159.03, 157.31, 152.72, 134.16, 126.50, 125.35, 120.35, 117.32, 99.83, 17.23 ppm. HR‐ESI‐MS (m/z): calcd for C_14_H_10_N_2_O_4_ [M + H]^+^: 271.0713, found 271.0719.

#### 4.6.7 3‐(1*H*‐Imidazole‐1‐Carbonyl)‐4‐Methyl‐2*H*‐Chromen‐2‐One (**IIa7**)

Light yellow solid powder, 54% yield; melting point: 135.4°C–141.3°C; **FT‐IR (KBr, cm^−^^1^)**: 3135 cm^−^^1^ (C─H arom.), 1716 cm^−^^1^ (C═O), 1601 cm^−^^1^ (C═N), 1562, 1523 cm^−^^1^ (C═C), 1317 cm^−^^1^ (C─N), 1252 cm^−^^1^ (C─O), 953 cm^−^^1^ (lactone skeleton), 905 cm^−^^1^ (C─H imidazole), 798, 761 cm^−^^1^ (C─H arom.), 633 cm^−^^1^ (ring deformation) (Figure S13); ^1^H NMR (600 MHz, CDCl_3_) δ 8.05 (s, 1H, imidazole‐H‐2′), 7.77 (d, *J* = 8.0 Hz, 1H, H‐5), 7.73 – 7.68 (m, 1H, H‐6), 7.49 (s, 1H, imidazole‐H‐5′), 7.47–7.42 (m, 2H, H‐7, H‐8), 7.15 (s, 1H, imidazole‐H‐4′), 2.50 (s, 3H, 4‐CH_3_) ppm (Figure S14); ^13^C NMR (151 MHz, CDCl_3_) δ 161.53, 157.53, 153.34, 137.09, 134.01, 131.80, 125.70, 125.31, 120.45, 118.68, 117.60, 116.42, 16.45 ppm. HR‐ESI‐MS (m/z):calcd for C_14_H_10_N_2_O_3_ [M + H]^+^: 255.0764, found 255.0751.

#### 4.6.8 *N*‐Benzyl‐7‐Methoxy‐4‐Methyl‐2‐Oxo‐2*H*‐Chromene‐3‐Carboxamide (**IIb1**)

White solid, 57% yield; melting point: 139.6°C–142.7°C; **FT‐IR (KBr, cm^−^^1^)**: 3269 cm^−^^1^ (N─H), 3072 cm^−^^1^ (C─H), 3000, 2846 cm^−^^1^ (C─H), 1708 cm^−^^1^ (C═O), 1621 cm^−^^1^ (C═O), 1562 cm^−^^1^ (amide II), 1509 cm^−^^1^ (C═C), 1229, 1094, 1026 cm^−^^1^ (C─O), 974 cm^−^^1^ (lactone), 884, 783, 710 cm^−^^1^ (C─H) (Figure S15); ^1^H NMR (400 MHz, CDCl_3_) δ 7.88 (s, 1H, −NH), 7.59 (d, *J* = 8.9 Hz, 1H, H‐5), 7.31 – 7.21 (m, 4H, H‐2′, H‐3′, H‐5′, H‐6′), 7.18 (d, *J* = 8.7 Hz, 1H, H‐4′), 6.81 (dd, *J* = 8.9, 2.6 Hz, 1H, H‐6), 6.69 (d, *J* = 2.6 Hz, 1H, H‐8), 4.53 (d, *J* = 5.8 Hz, 2H, −CH_2_NH−), 3.79 (s, 3H, 7‐OCH_3_), 2.66 (s, 3H, 4‐CH_3_) ppm (Figure S16); ^13^C NMR (101 MHz, CDCl_3_) δ 164.39, 163.85, 161.02, 156.97, 154.57, 138.24, 128.80, 127.78, 127.48, 127.29, 116.56, 114.01, 113.51, 100.41, 56.01, 43.90, 16.95 ppm. HR‐ESI‐MS (m/z): calcd for C_19_H_17_NO_4_ [M + H]^+^: 324.1230, found 324.1236.

#### 4.6.9 *N*‐(4‐Chlorobenzyl)‐7‐Methoxy‐4‐Methyl‐2‐Oxo‐2*H*‐Chromene‐3‐Carboxamide (**IIb2**)

White solid powder, 53% yield; melting point: 160.5°C–163.6°C; **FT‐IR (KBr, cm^−^^1^)**: 3332 cm^−^^1^ (N─H), 2929, 2846 cm^−^^1^ (C─H), 1697, 1615 cm^−^^1^ (C═O), 1541, 1494 cm^−^^1^ (C═C), 1223, 1019 cm^−^^1^ (C─O), 983 cm^−^^1^ (lactone), 853, 812, 720 cm^−^^1^ (C─H), 640 cm^−^^1^ (C─Cl) (Figure S17); ^1^H NMR (400 MHz, CDCl_3_) δ 8.11 (s, 1H, −NH), 7.70 (d, *J* = 9.0 Hz, 1H, H‐5), 7.31 (m, 4H, H‐2′, H‐3′, H‐5′, H‐6′), 6.92 (dd, *J* = 9.0, 2.6 Hz, 1H, H‐6), 6.79 (d, *J* = 2.5 Hz, 1H, H‐8), 4.59 (d, *J* = 5.8 Hz, 2H, −CH_2_NH−), 3.89 (s, 3H, 7‐OCH_3_), 2.77 (s, 3H, 4‐CH_3_) ppm (Figure S18); ^13^C NMR (101 MHz, CDCl_3_) δ 164.49, 163.98, 161.17, 157.54, 154.59, 136.91, 133.22, 129.10, 128.91, 127.38, 116.12, 114.03, 113.62, 100.42, 56.05, 43.19, 17.00 ppm. HR‐ESI‐MS (m/z): calcd for C_19_H_16_ClNO_4_ [M + H]^+^: 358.0841, found 358.0846.

#### 4.6.10 7‐Methoxy‐N‐(4‐Methoxybenzyl)‐4‐Methyl‐2‐Oxo‐2H‐Chromene‐3‐Carboxamide (**IIb3**)

Light orange solid powder, 57% yield; melting point: 158.7°C–161.2°C; **FT‐IR (KBr, cm^−^^1^)**: 3347 cm^−^^1^ (N─H), 2967, 2929, 2835 cm^−^^1^ (C─H), 1683 cm^−^^1^ (C═O), 1613 cm^−^^1^ (C═C), 1515 cm^−^^1^ (arom.), 1235 cm^−^^1^ (C─O─C), 1032 cm^−^^1^ (C─O), 987, 950 cm^−^^1^ (lactone), 861, 806 cm^−^^1^ (C─H), 720 cm^−^^1^ (arom.) (Figure S19); ^1^H NMR (400 MHz, CDCl_3_) δ 7.85 (s, 1H, −NH), 7.68 (d, *J* = 9.0 Hz, 1H, H‐5), 7.30 (d, *J* = 8.8 Hz, 2H, H‐2′, H‐6′), 6.93 – 6.85 (m, 3H, H‐6, H‐3′, H‐5′), 6.79 (d, *J* = 2.6 Hz, 1H, H‐8), 4.56 (d, *J* = 5.7 Hz, 2H, −CH_2_NH−), 3.88 (s, 3H, 7‐OCH_3_), 3.79 (s, 3H, 4′‐OCH_3_), 2.75 (s, 3H, 4‐CH_3_) ppm (Figure S20); ^13^C NMR (101 MHz, CDCl_3_) δ 164.28, 163.83, 160.98, 159.07, 156.78, 154.59, 130.35, 129.18, 127.27, 116.71, 114.21, 114.02, 113.49, 100.44, 56.02, 55.43, 43.42, 16.94 ppm. HR‐ESI‐MS (m/z): calcd for C_20_H_19_NO_5_ [M + H]^+^: 354.1336, found 354.1341.

#### 4.6.11 *N*‐(3,5‐Bis(Trifluoromethyl)Benzyl)‐7‐Methoxy‐4‐Methyl‐2‐Oxo‐2*H*‐Chromene‐3‐Carboxamide (**IIb4**)

White solid, 62% yield; melting point: 197.2°C–200.1°C; **FT‐IR (KBr, cm^−^^1^)**: 3285 cm^−^^1^ (N─H), 2849 cm^−^^1^ (C─H), 1726 cm^−^^1^ (C═O), 1632 cm^−^^1^ (C═O), 1385, 1352 cm^−^^1^ (CF_3_), 1276 cm^−^^1^ (C─F), 1188, 1124 cm^−^^1^ (C─F), 1034 cm^−^^1^ (C─O), 970 cm^−^^1^ (lactone), 884, 872, 835 cm^−^^1^ (C─H) (Figure S21); ^1^H NMR (400 MHz, CDCl_3_) δ 8.47 (s, 1H, −NH), 7.84 (s, 2H, H‐2′, H‐6′), 7.79–7.68 (m, 2H, H‐5, H‐4′), 6.93 (d, *J* = 9.0 Hz, 1H, H‐6), 6.80 (s, 1H, H‐8), 4.73 (d, *J* = 6.3 Hz, 2H, −CH_2_NH−), 3.88 (s, 3H, 7‐OCH_3_), 2.80 (s, 3H, 4‐CH_3_) ppm (Figure S22); ^13^C NMR (101 MHz, CDCl_3_) δ 164.21, 163.22, 161.91, 153.54, 152.87, 139.26, 129.40, 129.34, 129.14, 129.08, 127.01, 126.16, 126.12, 121.63, 114.93, 114.49, 110.71, 101.43, 55.66, 44.04, 16.96 ppm. HR‐ESI‐MS (m/z): calcd for C_21_H_15_F_6_NO_4_ [M + H]^+^: 460.0978, found 460.0983.

#### 4.6.12 7‐Methoxy‐4‐Methyl‐2‐Oxo‐*N*‐(Thiazol‐2‐yl)‐2*H*‐Chromene‐3‐Carboxamide (**IIb5**)

Light yellow solid powder, 54% yield; melting point: 233.9°C–235.9°C; **FT‐IR (KBr, cm^−^^1^)**: 3240 cm^−^^1^ (N─H), 2940, 2844 cm^−^^1^ (C─H), 1709 cm^−^^1^ (C═O), 1613 cm^−^^1^ (C═N), 1252 cm^−^^1^ (C─N), 1020 cm^−^^1^ (C─O), 948 cm^−^^1^ (lactone), 890, 855 cm^−^^1^ (C─H), 728, 685 cm^−^^1^ (C─S), 609 cm^−^^1^ (C─S) (Figure S23); ^1^H NMR (400 MHz, DMSO‐*d*_6_) δ 12.54 (s, 1H, −NH), 7.83 (d, *J* = 8.9 Hz, 1H, H‐5), 7.54 (d, *J* = 3.5 Hz, 1H, thiazole‐H‐4′), 7.32 (d, *J* = 3.6 Hz, 1H, thiazole‐H‐5′), 7.09 – 7.01 (m, 2H, H‐6, H‐8), 3.90 (s, 3H, 7‐OCH_3_), 2.41 (s, 3H, 4‐CH_3_) ppm (Figure S24); ^13^C NMR (101 MHz, DMSO‐*d*_6_) δ 163.30, 162.65, 158.28, 157.55, 154.37, 151.17, 138.01, 127.65, 119.02, 114.20, 112.99, 112.35, 100.83, 56.19, 15.95 ppm. HR‐ESI‐MS (m/z): calcd for C_15_H_12_N_2_O_4_S [M + H]^+^: 317.0591, found 317.0596.

#### 4.6.13 *N*‐(Isoxazol‐3‐yl)‐7‐Methoxy‐4‐Methyl‐2‐Oxo‐2*H*‐Chromene‐3‐Carboxamide (**IIb6**)

White flake solid, 68% yield; melting point: 218.8°C–223.8°C; **FT‐IR (KBr, cm^−^^1^)**: 3332 cm^−^^1^ (N─H), 2944, 2846 cm^−^^1^ (C─H), 1685 cm^−^^1^ (C═O), 1617 cm^−^^1^ (C═N), 1465 cm^−^^1^ (arom.), 1264 cm^−^^1^ (C─N), 1026 cm^−^^1^ (C─O), 917 cm^−^^1^ (N─O), 874, 818 cm^−^^1^ (C─H), 783 cm^−^^1^ (arom.) (Figure S25); ^1^H NMR (400 MHz, DMSO‐*d*_6_) δ 11.53 (s, 1H, −NH), 8.87 (s, 1H, isoxazole‐H‐5′), 7.82 (d, *J* = 8.8 Hz, 1H, H‐5), 7.10 – 6.96 (m, 3H, H‐6, H‐8, isoxazole‐H‐4′), 3.89 (s, 3H, 7‐OCH_3_), 2.42 (s, 3H, 4‐CH_3_) ppm (Figure S26); ^13^C NMR (101 MHz, DMSO‐*d*_6_) δ 163.12, 162.96, 160.58, 158.21, 157.19, 154.24, 150.33, 127.51, 120.03, 112.87, 112.30, 100.77, 99.18, 56.11, 15.83 ppm. HR‐ESI‐MS (m/z): calcd for C_15_H_12_N_2_O_5_ [M + H]^+^: 301.0819, found 301.0824.

#### 4.6.14 3‐(1*H*‐Imidazole‐1‐Carbonyl)‐7‐Methoxy‐4‐Methyl‐2*H*‐Chromen‐2‐One (**IIb7**)

Light yellow solid powder, 63% yield; melting point: 165.7°C–171.2°C; **FT‐IR (KBr, cm^−^^1^)**: 3074 cm^−^^1^ (C─H), 2942, 2846 cm^−^^1^ (C─H), 1716 cm^−^^1^ (C═O), 1609 cm^−^^1^ (C═N), 1262 cm^−^^1^ (C─O), 1013 cm^−^^1^ (C─O), 954 cm^−^^1^ (lactone), 907, 868 cm^−^^1^ (C─H), 677 cm^−^^1^ (arom.) (Figure S27); ^1^H NMR (400 MHz, CDCl_3_) δ 8.03 (s, 1H, imidazole‐H‐2′), 7.67 – 7.61 (m, 1H, H‐5), 7.48 (s, 1H, imidazole‐H‐5′), 7.12 (s, 1H, imidazole‐H‐4′), 6.96 (d, *J* = 8.9 Hz, 1H, H‐6), 6.88 (d, *J* = 1.6 Hz, 1H, H‐8), 3.93 (s, 3H, 7‐OCH_3_), 2.44 (br s, *J* = 1.6 Hz, 3H, 4‐CH_3_) ppm (Figure S28); ^13^C NMR (101 MHz, CDCl_3_) δ 164.61, 162.04, 158.14, 155.56, 154.02, 137.31, 131.72, 127.01, 117.10, 116.60, 113.87, 112.32, 101.14, 56.09, 16.54 ppm. HR‐ESI‐MS (m/z): calcd for C_15_H_12_N_2_O_4_ [M + H]^+^: 285.0870, found 285.0875.

#### 4.6.15 3‐Amino‐7‐Hydroxy‐4H‐Thieno[3,4‐c]Chromen‐4‐One (**III**)

Purple solid powder, 54% yield; melting point: 266.7°C–270.9°C; **FT‐IR (KBr, cm^−^^1^)**: 3400, 3306, 3180 cm^−^^1^ (N─H/O─H), 1675 cm^−^^1^ (C═O), 1607 cm^−^^1^ (C═C), 1502 cm^−^^1^ (arom.), 1278, 1159 cm^−^^1^ (C─O), 976 cm^−^^1^ (lactone), 849, 810, 769 cm^−^^1^ (C─H), 666, 621 cm^−^^1^ (C─S) (Figure S29); ^1^H NMR (400 MHz, DMSO‐*d*_6_) δ 9.96 (s, 1H, 7‐OH), 7.71 (s, 2H, −NH_2_), 7.66 (d, *J* = 8.5 Hz, 1H, H‐5), 6.66 (dd, *J* = 8.5, 2.4 Hz, 1H, H‐6), 6.59 (s, 1H, thiophene‐H‐2′), 6.57 (d, *J* = 2.4 Hz, 1H, H‐8) ppm (Figure S30); ^13^C NMR (101 MHz, DMSO‐*d*_6_) δ 163.31, 158.41, 157.25, 152.74, 137.74, 127.21, 114.17, 112.17, 106.12, 104.28, 101.15 ppm. HR‐ESI‐MS (m/z): calcd for C_11_H_7_NO_3_S [M + H]^+^: 234.0219, found 234.0227.

#### 4.6.16 3‐Amino‐4‐Oxo‐4*H*‐Thieno[3,4‐c]Chromen‐7‐yl‐Trifluoromethanesulfonate (**IVa**)

Light yellow solid, 21% yield; melting point: 94.8°C–96.2°C; **FT‐IR (KBr, cm^−^^1^)**: 3429, 3300, 3193 cm^−^^1^ (NH_2_), 1712 cm^−^^1^ (C═O), 1609 cm^−^^1^ (C═C), 1391 cm^−^^1^ (SO_2_), 1221 cm^−^^1^ (SO_2_), 1139, 1118, 1098 cm^−^^1^ (C─F), 1057 cm^−^^1^ (S─O), 970 cm^−^^1^ (skeleton), 880 cm^−^^1^ (C─H) (Figure S31); ^1^H NMR (400 MHz, CDCl_3_) δ 7.73 (d, *J* = 8.7 Hz, 1H, H‐5), 7.17 (d, *J* = 2.5 Hz, 1H, H‐8), 7.12 (dd, *J* = 8.7, 2.5 Hz, 1H, H‐6), 6.54 (s, 1H, thiophene‐H‐2′), 6.28 (s, 2H, −NH_2_) ppm (Figure S32); ^13^C NMR (101 MHz, CDCl_3_) δ 165.24, 159.26, 152.03, 148.99, 130.69, 124.81, 118.52, 117.49, 111.31, 100.53, 98.49 ppm. HR‐ESI‐MS (m/z): calcd for C_12_H_6_F_3_NO_5_S_2_ [M + H]^+^: 365.9712, found 365.9730.

#### 4.6.17 3‐Amino‐4‐Oxo‐4H‐Thieno[3,4‐c]Chromen‐7‐yl Isobutyl Carbonate (**IVb**)

Brown solid, 20% yield; melting point: 107.5°C–111.3°C; **FT‐IR (KBr, cm^−^^1^)**: 3458, 3318 cm^−^^1^ (NH_2_), 2969, 2929, 2881 cm^−^^1^ (C─H), 1761 cm^−^^1^ (C═O), 1704 cm^−^^1^ (C═O), 1225 cm^−^^1^ (C─O), 1131 cm^−^^1^ (C─O─C), 986 cm^−^^1^ (skeleton), 935 cm^−^^1^ (skeleton), 888, 767 cm^−^^1^ (C─H), 736, 695 cm^−^^1^ (heterocycle) (Figure S33); ^1^H NMR (400 MHz, CDCl_3_) δ 7.64 (d, *J* = 8.5 Hz, 1H), 7.64 (d, *J* = 8.5 Hz, 1H, H‐5), 7.07 (d, *J* = 2.4 Hz, 1H, H‐8), 7.03 (dd, *J* = 8.5, 2.4 Hz, 1H, H‐6), 6.44 (s, 1H, thiophene‐H‐2′), 6.24 (s, 2H, −NH_2_), 4.06 (d, *J* = 6.7 Hz, 2H, −O‐CH_2_−), 2.07 (dp, *J* = 13.4, 6.7 Hz, 1H, −CH(CH_3_)_2_), 1.01 (d, *J* = 6.8 Hz, 6H, −(CH_3_)_2_) ppm (Figure S34); ^13^C NMR (101 MHz, CDCl_3_) δ 165.14, 159.86, 153.63, 151.90, 151.24, 131.31, 124.07, 117.44, 116.20, 110.81, 100.63, 97.16, 75.28, 27.92, 19.02 ppm. HR‐ESI‐MS (m/z): calcd for C_16_H_15_NO_5_S [M + H]^+^: 334.0744, found 334.0754.

#### 4.6.18 3‐Amino‐7‐((2‐Methylallyl)oxy)‐4*H*‐Thieno[3,4‐c]Chromen‐4‐One (**IVc**)

Brown solid, 25% yield; melting point: 113.2°C–119.7°C; **FT‐IR (KBr, cm^−^^1^)**: 3441, 3349 cm^−^^1^ (NH_2_), 3098 cm^−^^1^ (C─H), 2919 cm^−^^1^ (C─H), 1712 cm^−^^1^ (C═O), 1615 cm^−^^1^ (C═C), 1266 cm^−^^1^ (C─O─C), 1161, 1013 cm^−^^1^ (C─O), 970, 888 cm^−^^1^ (═CH_2_), 804, 765 cm^−^^1^ (C─H) (Figure S35); ^1^H NMR (400 MHz, CDCl_3_) δ 7.62 (d, *J* = 8.5 Hz, 1H, H‐5), 6.90 – 6.79 (m, 2H, H‐6, H‐8), 6.39 (s, 1H, thiophene‐H‐2′), 6.29 (s, 2H, −NH_2_), 5.21 – 5.04 (m, 2H, = CH_2_), 4.52 (s, 2H, −O‐CH_2_−), 1.90 (s, 3H, = C(CH_3_)) ppm (Figure S36); ^13^C NMR (101 MHz, CDCl_3_) δ 164.78, 160.51, 159.72, 152.62, 140.41, 132.32, 124.17, 113.36, 112.58, 111.37, 102.90, 101.13, 94.92, 72.17, 19.49 ppm. HR‐ESI‐MS (m/z): calcd for C_15_H_13_NO_3_S [M + H]^+^: 288.0689, found 288.0713.

#### 4.6.19 3‐Amino‐7‐(Isopentyloxy)‐4H‐Thieno[3,4‐c]Chromen‐4‐One (**IVd**)

Brown solid powder, 40% yield; melting point: 115.0°C–121.1°C; **FT‐IR (KBr, cm^−^^1^)**: 3437, 3335 cm^−^^1^ (NH_2_), 2955, 2874 cm^−^^1^ (C─H), 1708 cm^−^^1^ (C═O), 1621, 1582 cm^−^^1^ (C═C), 1266 cm^−^^1^ (C─O─C), 1147, 1099, 1055 cm^−^^1^ (C─O) (Figure S37); ^1^H NMR (400 MHz, CDCl_3_) δ 7.54 (d, *J* = 8.5 Hz, 1H, H‐5), 6.73 (m, 2H, H‐6, H‐8), 6.28 (br m, overlap, 3H, thiophene‐H‐2′, −NH_2_), 4.00 (t, *J* = 6.7 Hz, 2H, −O‐CH_2_−), 1.83 (hept, *J* = 6.7 Hz, 1H, −CH−), 1.68 (m, 2H, −CH_2_−), 0.97 (d, *J* = 6.6 Hz, 6H, −(CH_3_)_2_) ppm (Figure S38); ^13^C NMR (101 MHz, CDCl_3_) δ 164.84, 160.55, 160.13, 152.64, 132.32, 124.13, 112.34, 111.03, 102.41, 101.04, 94.73, 66.92, 37.90, 25.17, 22.69 ppm. HR‐ESI‐MS (m/z): calcd for C_16_H_17_NO_3_S [M + H]^+^: 304.1002, found 304.1009.

### 4.7 Evaluation of Antifungal Activity Against Plant Pathogenic Fungi

The test fungal strains included *Fusarium solani* (*F. solani)*, *Leptosphaeria biglobosa* (*L. biglobosa*), *Valsa mali* (*V. mali*), and *Botrytis cinerea* (*B. cinerea*), provided by the Microbiology Laboratory of Shaanxi University of Science and Technology. All strains were cultured on Potato Dextrose Agar (PDA) medium at 25°C. The antifungal activity of coumarin derivatives was evaluated using the mycelial growth rate method [51, 52], with the inhibition rate serving as the assessment criterion. Azoxystrobin (AZ) was used as the positive control, while PDA medium without any compound served as the negative control. Preliminary screening was performed at a concentration of 100 µg/mL. The inhibition rate was calculated using the following formula:

$$\text{Inhibition rate}\left(\%\right)=\frac{D_{0}-D_{1}}{D_{1}-0.5}\times 100\%$$

where *D*_0_ represents the colony diameter (cm) of the negative control group, and *D*_1_ represents the colony diameter (cm) of the compound‐treated group. Antifungal inhibition rates (%) are presented as the mean of three independent replicates.

The compound exhibiting the most potent antifungal activity was selected for determination of its half‐maximal effective concentration (EC_50_) [53]. A series of concentration gradients (200, 100, 50, 25, 12.5, and 6.25 µg/mL) was prepared and incorporated into PDA medium to generate compound‐amended plates. When the mycelium in the negative control group had fully colonized the plates, the colony diameters at each concentration were measured. Three replicates were maintained for each concentration, and the mean values were calculated. The inhibition rates were used to establish a toxicity regression equation to determine the EC_50_.

### 4.8 Evaluation of Synergistic Effects Between **IVd** and Positive Control Agents

Azoxystrobin (AZ) and fluconazole (FLC) were selected as positive control agents. The EC_50_ values of **IVd** and these positive controls were determined as described in Section 2.3. The theoretical half‐maximal effective concentrations (EC_50_, Exp) for combinations of **IVd** with each positive control at mass ratios of 1:2, 1:1, and 2:1 were calculated according to Wadley's method. The calculation formula is as follows:

$$EC_{50}\left(Exp\right)=\frac{A+B}{\frac{A}{EC_{50}\left(A\right)}+\frac{B}{EC_{50}\left(B\right)}}$$

where *A* represents the mass percentage (%) of the positive control agent; *B* represents the mass percentage (%) of the test compound (**IVd**); EC_50_(*A*) represents the half‐maximal effective concentration (µg/mL) of the positive control agent; and EC_50_(*B*) represents the half‐maximal effective concentration (µg/mL) of the test compound.

The observed half‐maximal effective concentrations (EC_50_, Obs) of the drug combinations at each mass ratio (1:2, 1:1, and 2:1) were experimentally determined. The synergistic ratio (SR) was calculated for each combination [54] as follows: SR = EC_50_(Exp)/EC_50_(Obs). An SR value > 1.5 indicates synergism, an SR < 0.5 indicates antagonism, and values between 0.5 and 1.5 indicate additive effects.

$$SR=\frac{EC_{50}\left(Exp\right)}{EC_{50}\left(Obs\right)}$$

### 4.9 Effects of **IVd** Treatment on Membrane Integrity and Lipid Peroxidation in *V. mali*

The effect of **IVd** treatment on the membrane permeability of *V. mali* hyphae was assessed by measuring electrolyte leakage [29]. Mycelial plugs (5 mm diameter) of *V. mali* were inoculated into potato dextrose broth (PDB) containing **IVd** (80 µg/mL) and incubated at 25°C with shaking at 160 rpm for 72 h. Three independent replicates were prepared for each condition. Samples were collected at 72, 84, 96, 108, and 120 h, followed by filtration and centrifugation to obtain the supernatant. The conductivity of the supernatant was measured using a DDSJ‐308A conductivity meter (Beijing Zhongyi Kexin Technology Co., Ltd.).

The extent of lipid peroxidation induced by **IVd** treatment in *V. mali* was evaluated by quantifying the malondialdehyde (MDA) content [29, 55]. Hyphae were cultivated under the same conditions described above. Mycelia were harvested at 72, 84, 96, 108, and 120 h, and the MDA content was determined according to the manufacturer's instructions provided with the MDA assay kit.

### 4.10 Scanning Electron Microscopy (SEM) Analysis of Hyphal Morphology

Scanning electron microscopy (SEM; FEI Quanta 450 environmental SEM, USA) was employed to examine the effects of compound **IVd** on the hyphal morphology of *V. mali*. Mycelia from plates treated with 80 µg/mL **IVd** were collected and processed according to a previously described method [56]. Briefly, samples were fixed, rinsed, dehydrated, and subsequently freeze‐dried for 12 h using a vacuum freeze‐dryer (FD‐ID‐50, Shanghai Bilang Instrument Manufacturing Co., Ltd.). The dried samples were then mounted on stubs using conductive adhesive, sputter‐coated with gold, and observed under the SEM at a magnification of ×3000 [57].

### 4.11 Molecular Docking

The crystal structures of CYP51 (PDB: 5FSA), CYP51B (PDB: 6CR2), and SDH (PDB: 2FBW) were retrieved from the Protein Data Bank (PDB). Ligands were protonated at pH 7.4, and their 3D structures were generated using Open Babel [58]. AutoDock Tools (ADT3) were employed to prepare and parameterize the receptors and ligands. Docking grids were generated with AutoGrid, and docking simulations were performed using AutoDock Vina (v1.2.0) [59, 60]. The optimal binding pose was subsequently selected for interaction analysis. Protein–ligand interactions were visualized with PyMOL.

### 4.12 Statistical Analysis

All experiments were conducted in triplicate. Data are presented as the mean ± standard deviation (SD). Statistical significance was assessed using one‐way analysis of variance (ANOVA) via SPSS software (Version 27, IBM Corp., USA), followed by post hoc multiple comparisons using the least significant difference (LSD) test. Differences were deemed statistically significant at *p <* 0.05 and highly significant at *p <* 0.01.

## Author Contributions

**Xiangna Chang**: conceptualization, methodology, design, writing – original draft preparation, review and editing, and funding acquisition. **Qiang Du**: investigation, data processing, and analysis. **Xiaoying Ma**: analysis, data curation, and writing – original draft preparation. **Jundi Zhang**: analysis, validation, and writing. All authors have read and agreed to the published version of the manuscript.

## Funding

This work was jointly supported by the National Natural Science Foundation of China (No. 32572709), the Project from Weiyang Technology Bureau of Xi'an (No. 202415), and the Doctoral Research Start‐up Fund of Shaanxi University of Science and Technology (No. 2022BJ‐31)

## Conflicts of Interest

The authors declare no conflicts of interest.

## Supporting information




**Supporting File**: cbdv71773‐sup‐0001‐SuppMat.docx
Supporting information for this article is available on the WWW under https://doi.org/10.1002/MS‐number.

## Data Availability

The data that supports the findings of this study are available in the supplementary material of this article.
