# Supplementary material for: Coumarin‐Based Antifungal Leads: Design, Synthesis, Mechanistic Insights, and Synergistic Activity
Source: Chem Biodivers. 2026 Sep 30;23(10):e71773. doi: 10.1002/cbdv.71773 (PMC13626469; doi:10.1002/cbdv.71773)
Supplement: Supplementary file 1 [file CBDV-23-e71773-s001.docx]

**Supporting Information**

**Coumarin-Based Antifungal Leads: Design, Synthesis, Mechanistic Insights, and Synergistic Activity**

Xiangna Chang (s),^a^ Qiang Du (s),^a^ Xiaoying Ma (s),^a^ Jundi Zhang (s),^a*^

^[a]^School of Food Science and Engineering, Shaanxi University of Science & Technology, Xi’an 710021, China;

*changxiangna@sust.edu.cn

**Chemical characterization of compounds IIa1-IIa7**

***N*-benzyl-4-methyl-2-oxo-2*H*-chromene-3-carboxamide (IIa1):** White solid powder,72% yield; Melting Point: 146.6-149.6 °C; **FT-IR (KBr, cm^-1^):** 3359 cm⁻¹ (N–H), 3074, 3041 cm⁻¹ (C–H), 2924 cm⁻¹ (C–H), 1695 cm⁻¹ (C=O), 1658 cm⁻¹ (C=O), 1609 cm⁻¹ (C=C), 1527 cm⁻¹ (amide II), 1252 cm⁻¹ (C–O–C), 1186, 1143 cm⁻¹ (C–O), 968 cm⁻¹ (lactone), 759 cm⁻¹ (C–H), 726, 693 cm⁻¹ (C–H), 658 cm⁻¹ (aromatic); ^1^H NMR (600 MHz, CDCl_3_) δ 7.79 (d, *J* = 8.2 Hz, 1H, H‑5), 7.72 (s, 1H, −NH), 7.60 (t, *J* = 7.7 Hz, 1H, H‑6), 7.39 (d, *J* = 7.1 Hz, 2H, H‑2′, H‑6′), 7.35 (t, *J* = 7.8 Hz, 4H, H‑7, H‑8, H‑3′, H‑5′, overlapping), 7.29 (d, *J* = 7.3 Hz, 1H, H‑4′), 4.65 (d, *J* = 5.8 Hz, 2H, −CH₂NH−), 2.77 (s, 3H, 4‑CH₃) ppm; ^13^C NMR (151 MHz, CDCl_3_) δ 164.05, 160.15, 155.31, 152.58, 138.00, 133.03, 128.79, 127.75, 127.52, 125.91, 124.89, 120.52, 120.22, 117.01, 43.90, 16.71ppm. HR- ESI -MS (m/z):calcd for C_18_H_15_NO_3_ [M + H]^+^: 294.1125, found 294.1114.

**Figure S1:** Infrared spectrum ʋmax (KBr, cm^-1^) of compound **IIa1**

**Figure S1**: ^1^H NMR spectrum (400 MHz, CDCl_3_) of compound **IIa1**

***N*-(4-chlorobenzyl)-4-methyl-2-oxo-2*H*-chromene-3-carboxamide (IIa2):** White solid powder,69% yield; Melting Point: 170.9-171.4 °C; **FT-IR (KBr, cm⁻¹):** 3361 cm⁻¹ (N–H), 3047 cm⁻¹ (C–H), 2913 cm⁻¹ (C–H), 1699 cm⁻¹ (C=O), 1609 cm⁻¹ (C=C), 1527 cm⁻¹ (amide II), 1492 cm⁻¹ (aromatic), 1254 cm⁻¹ (C–O–C), 1179 cm⁻¹ (C–O), 1032 cm⁻¹ (C–O), 968 cm⁻¹ (lactone), 812 cm⁻¹ (C–H), 757 cm⁻¹ (C–H), 627 cm⁻¹ (C–Cl). ^1^H NMR (600 MHz, CDCl_3_) δ 7.85 (s, 1H, −NH), 7.80 (dd, *J* = 8.0, 1.6 Hz, 1H, H‑5), 7.63 – 7.59 (m, 1H, H‑6), 7.40 – 7.29 (m, 6H, H‑7, H‑8, H‑2′, H‑3′, H‑5′, H‑6′), 4.61 (d, *J* = 5.8 Hz, 2H, −CH₂NH−), 2.77 (s, 3H, 4‑CH₃) ppm; ^13^C NMR (101 MHz, CDCl_3_) δ 164.14, 160.43, 156.31, 152.61, 136.68, 133.32, 133.27, 129.12, 128.95, 126.05, 125.03, 120.33, 119.92, 117.10, 43.26, 16.87 ppm. HR- ESI -MS (m/z):calcd for C_18_H_14_ClNO_3_ [M + H]^+^: 328.0735, found 328.0740.

**Figure S3**: Infrared spectrum ʋmax (KBr, cm^-1^) of compound **IIa2**

**Figure S4**: ^1^H NMR spectrum (400 MHz, CDCl_3_) of compound **IIa2**

***N*-(4-methoxybenzyl)-4-methyl-2-oxo-2*H*-chromene-3-carboxamide (IIa3):** White solid powder,74% yield; Melting Point: 185.8-187.3 °C; **FT-IR (KBr, cm⁻¹):** 3349, 3252 cm⁻¹ (N–H), 3061 cm⁻¹ (C–H), 2994, 2946 cm⁻¹ (C–H), 2844 cm⁻¹ (OCH₃), 1695 cm⁻¹ (C=O), 1638 cm⁻¹ (C=O), 1611 cm⁻¹ (C=C), 1519 cm⁻¹ (aromatic), 1246 cm⁻¹ (C–O–C), 1180 cm⁻¹ (C–O), 1032 cm⁻¹ (C–O), 968 cm⁻¹ (lactone), 812 cm⁻¹ (C–H), 759 cm⁻¹ (C–H). ^1^H NMR (600 MHz, CDCl_3_) δ 7.79 (d, *J* = 8.2 Hz, 1H, H‑5), 7.60 (t, *J* = 7.0 Hz, 1H, H‑6), 7.60 (s, 1H, −NH, overlapping with H‑6), 7.38 – 7.34 (m, 2H, H‑7, H‑8), 7.32 (d, *J* = 8.7 Hz, 2H, H‑2′, H‑6′), 6.89 (d, *J* = 8.7 Hz, 2H, H‑3′, H‑5′), 4.58 (d, *J* = 5.6 Hz, 2H, −CH₂NH−), 3.80 (s, 3H, 4′‑OCH₃), 2.77 (s, 3H, 4‑CH₃) ppm; ^13^C NMR (101 MHz, CDCl_3_) δ 163.93, 160.25, 159.13, 155.55, 152.65, 133.09, 130.11, 129.21, 125.96, 124.93, 120.48, 120.33, 117.09, 114.24, 55.44, 43.50, 16.80 ppm. HR- ESI -MS (m/z):calcd for C_19_H_17_NO_4_ [M + H]^+^: 346.1050, found 346.1057.

**Figure S5**: Infrared spectrum ʋmax (KBr, cm^-1^) of compound **IIa3**

**Figure S6**: ^1^H NMR spectrum (400 MHz, CDCl_3_) of compound **IIa3**

***N*-(3,5-bis(trifluoromethyl)benzyl)-4-methyl-2-oxo-2*H*-chromene-3-carboxamide (IIa4):** White solid powder,72% yield; Melting Point: 225.3-227.9 °C; **FT-IR (KBr, cm⁻¹):** 3320 cm⁻¹ (N–H), 3078 cm⁻¹ (C–H), 2934 cm⁻¹ (C–H), 1712 cm⁻¹ (C=O), 1661 cm⁻¹ (C=O), 1615 cm⁻¹ (C=C), 1379, 1357 cm⁻¹ (CF₃), 1281, 1168, 1120 cm⁻¹ (C–F), 977 cm⁻¹ (lactone), 753 cm⁻¹ (C–H), 679 cm⁻¹ (aromatic).^1^H NMR (600 MHz, CDCl_3_) δ 8.24 (s, 1H, −NH), 7.86 (s, 2H, H‑2′, H‑6′), 7.84 (dd, *J* = 8.1, 1.5 Hz, 1H, H‑5), 7.79 (s, 1H, H‑4′), 7.64 (ddd, *J* = 8.5, 7.3, 1.5 Hz, 1H, H‑6), 7.41 – 7.37 (m, 2H, H‑7, H‑8), 4.77 (d, *J* = 6.0 Hz, 2H, −CH₂NH−), 2.82 (s, 3H, 4‑CH₃) ppm; ^13^C NMR (151 MHz, CDCl_3_) δ 164.42, 160.65, 157.63, 152.51, 140.96, 133.47, 132.07, 131.84, 127.55, 126.11, 125.05, 121.38, 120.26, 118.84, 117.05, 42.89, 16.80 ppm. HR- ESI -MS (m/z):calcd for C_20_H_13_F_6_NO_3_ [M + H]^+^: 430.0872, found 430.0879.

**Figure S7:** Infrared spectrum ʋmax (KBr, cm^-1^) of compound **IIa4**

**Figure S8:** ^1^H NMR spectrum (500 MHz, CDCl_3_) of comp **IIa4**
**4-methyl-2-oxo-N-(thiazol-2-yl)-2H-chromene-3-carboxamide (IIa5):** Light yellow solid powder,64% yield; Melting Point: 242.8-246.4°C; **FT-IR (KBr, cm⁻¹):** 3228, 3201 cm⁻¹ (N–H), 1661 cm⁻¹ (C=O), 1605 cm⁻¹ (C=N/C=C), 1529, 1484 cm⁻¹ (aromatic), 1451 cm⁻¹ (C=C), 1194, 1163 cm⁻¹ (C–N/C–O), 972 cm⁻¹ (lactone), 761, 734 cm⁻¹ (C–H). ^1^H NMR (400 MHz, CDCl_3_) δ 12.03 (s, 1H, −NH), 7.90 (d, J = 6.9 Hz, 1H, H‑5), 7.68 (t, J = 8.5 Hz, 1H, H‑6), 7.41 (m, 3H, H‑7, H‑8, thiazole‑H‑4′, overlapping), 6.99 (d, J = 3.6 Hz, 1H, thiazole‑H‑5′), 2.95 (s, 3H, 4‑CH₃) ppm; ^13^C NMR (151 MHz, CDCl_3_) δ 164.70, 161.83, 160.19, 153.74, 152.09, 133.50, 129.01, 125.92, 124.39, 120.19, 117.67, 114.10, 112.57, 16.99 ppm. HR- ESI -MS (m/z):calcd for C_14_H_10_N_2_O_3_S [M + H]^+^: 287.0485, found 287.0490.

**Figure S9:** Infrared spectrum ʋmax (KBr, cm^-1^) of compound **IIa5**

**Figure S10**: ^1^H NMR spectrum (400 MHz, CDCl_3_) of compound **IIa5**

***N*-(isoxazol-3-yl)-4-methyl-2-oxo-2*H*-chromene-3-carboxamide (IIa 6):** White solid powder,61% yield; Melting Point: 217.6-225.9 °C; **FT-IR (KBr, cm⁻¹):** 3359, 3197 cm⁻¹ (N–H), 1708 cm⁻¹ (C=O), 1589 cm⁻¹ (C=N), 1535 cm⁻¹ (C=C), 1034 cm⁻¹ (N–O), 989, 958 cm⁻¹ (heterocycle), 761 cm⁻¹ (C–H arom.), 790, 710, 664 cm⁻¹ (ring deformation).  ^1^H NMR (400 MHz, CDCl_3_) δ 10.58 (s, 1H, −NH), 8.30 (s, 1H, isoxazole‑H‑5′), 7.86 (d, *J* = 7.8 Hz, 1H, H‑5), 7.65 (t, *J* = 7.8 Hz, 1H, H‑6), 7.38 (m, 2H, H‑7, H‑8), 7.11 (s, 1H, isoxazole‑H‑4′), 2.88 (s, 3H, 4‑CH₃) ppm; ^13^C NMR (101 MHz, CDCl_3_) δ 161.88, 160.70, 159.03, 157.31, 152.72, 134.16, 126.50, 125.35, 120.35, 117.32, 99.83, 17.23 ppm. HR- ESI -MS (m/z):calcd for C_14_H_10_N_2_O_4_ [M + H]^+^: 271.0713, found 271.0719.

**Figure S11:** Infrared spectrum ʋmax (KBr, cm^-1^) of compound **IIa6**

**Figure S12:** ^1^H NMR spectrum (500 MHz, CDCl_3_) of comp **IIa6**

**3-(1*H*-imidazole-1-carbonyl)-4-methyl-2*H*-chromen-2-one (IIa7):** Light yellow solid powder,54% yield; Melting Point: 135.4-141.3 °C; **FT-IR (KBr, cm⁻¹):** 3135 cm⁻¹ (C–H arom.), 1716 cm⁻¹ (C=O), 1601 cm⁻¹ (C=N), 1562, 1523 cm⁻¹ (C=C), 1317 cm⁻¹ (C–N), 1252 cm⁻¹ (C–O), 953 cm⁻¹ (lactone skeleton), 905 cm⁻¹ (C–H imidazole), 798, 761 cm⁻¹ (C–H arom.), 633 cm⁻¹ (ring deformation). ^1^H NMR (600 MHz, CDCl_3_) δ 8.05 (s, 1H, imidazole‑H‑2′), 7.77 (d, *J* = 8.0 Hz, 1H, H‑5), 7.73 – 7.68 (m, 1H, H‑6), 7.49 (s, 1H, imidazole‑H‑5′), 7.47 – 7.42 (m, 2H, H‑7, H‑8), 7.15 (s, 1H, imidazole‑H‑4′), 2.50 (s, 3H, 4‑CH₃) ppm; ^13^C NMR (151 MHz, CDCl_3_) δ 161.53, 157.53, 153.34, 137.09, 134.01, 131.80, 125.70, 125.31, 120.45, 118.68, 117.60, 116.42, 16.45 ppm. HR- ESI -MS (m/z):calcd for C_14_H_10_N_2_O_3_ [M + H]^+^: 255.0764, found 255.0751.

**Figure S13:** Infrared spectrum ʋmax (KBr, cm^-1^) of compound **IIa7**

**Figure S14:** ^1^H NMR spectrum (500 MHz, CDCl_3_) of comp **IIa7**

**Chemical characterization of compounds IIb1-IIb**

***N*-benzyl-7-methoxy-4-methyl-2-oxo-2*H*-chromene-3-carboxamide (IIba1):** White solid,57% yield; Melting Point: 139.6-142.7 °C; **FT-IR (KBr, cm⁻¹):** 3269 cm⁻¹ (N–H), 3072 cm⁻¹ (C–H), 3000, 2846 cm⁻¹ (C–H), 1708 cm⁻¹ (C=O), 1621 cm⁻¹ (C=O), 1562 cm⁻¹ (amide II), 1509 cm⁻¹ (C=C), 1229, 1094, 1026 cm⁻¹ (C–O), 974 cm⁻¹ (lactone), 884, 783, 710 cm⁻¹ (C–H). ^1^H NMR (400 MHz, CDCl_3_) δ 7.88 (s, 1H, −NH), 7.59 (d, *J* = 8.9 Hz, 1H, H‑5), 7.31 – 7.21 (m, 4H, H‑2′, H‑3′, H‑5′, H‑6′), 7.18 (d, *J* = 8.7 Hz, 1H, H‑4′), 6.81 (dd, *J* = 8.9, 2.6 Hz, 1H, H‑6), 6.69 (d, *J* = 2.6 Hz, 1H, H‑8), 4.53 (d, *J* = 5.8 Hz, 2H, −CH₂NH−), 3.79 (s, 3H, 7‑OCH₃), 2.66 (s, 3H, 4‑CH₃) ppm; ^13^C NMR (101 MHz, CDCl_3_) δ 164.39, 163.85, 161.02, 156.97, 154.57, 138.24, 128.80, 127.78, 127.48, 127.29, 116.56, 114.01, 113.51, 100.41, 56.01, 43.90, 16.95 ppm. HR- ESI -MS (m/z):calcd for C_19_H_17_NO_4_ [M + H]^+^: 324.1230, found 324.1236.

**Figure S15:** Infrared spectrum ʋmax (KBr, cm^-1^) of compound **IIb1**

**Figure S16**: ^1^H NMR spectrum (400 MHz, CDCl_3_) of compound **IIb1**

***N*-(4-chlorobenzyl)-7-methoxy-4-methyl-2-oxo-2*H*-chromene-3-carboxamide (IIb2):** White solid powder,53% yield; Melting Point: 160.5-163.6 °C; **FT-IR (KBr, cm⁻¹):** 3332 cm⁻¹ (N–H), 2929, 2846 cm⁻¹ (C–H), 1697, 1615 cm⁻¹ (C=O), 1541, 1494 cm⁻¹ (C=C), 1223, 1019 cm⁻¹ (C–O), 983 cm⁻¹ (lactone), 853, 812, 720 cm⁻¹ (C–H), 640 cm⁻¹ (C–Cl). ^1^H NMR (400 MHz, CDCl_3_) δ 8.11 (s, 1H, −NH), 7.70 (d, *J* = 9.0 Hz, 1H, H‑5), 7.31 (m, 4H, H‑2′, H‑3′, H‑5′, H‑6′), 6.92 (dd, *J* = 9.0, 2.6 Hz, 1H, H‑6), 6.79 (d, *J* = 2.5 Hz, 1H, H‑8), 4.59 (d, *J* = 5.8 Hz, 2H, −CH₂NH−), 3.89 (s, 3H, 7‑OCH₃), 2.77 (s, 3H, 4‑CH₃) ppm; ^13^C NMR (101 MHz, CDCl_3_) δ 164.49, 163.98, 161.17, 157.54, 154.59, 136.91, 133.22, 129.10, 128.91, 127.38, 116.12, 114.03, 113.62, 100.42, 56.05, 43.19, 17.00 ppm. HR- ESI -MS (m/z):calcd for C_19_H_16_ClNO_4_ [M + H]^+^: 358.0841, found 358.0846.

**Figure S17:** Infrared spectrum ʋmax (KBr, cm^-1^) of compound **IIb2**

**Figure S18**: ^1^H NMR spectrum (400 MHz, CDCl_3_) of compound **IIb2**

**7-methoxy-N-(4-methoxybenzyl)-4-methyl-2-oxo-2H-chromene-3-carboxamide (IIb3):** Light orange solid powder,57% yield; Melting Point: 158.7-161.2 °C; **FT-IR (KBr, cm⁻¹):** 3347 cm⁻¹ (N–H), 2967, 2929, 2835 cm⁻¹ (C–H), 1683 cm⁻¹ (C=O), 1613 cm⁻¹ (C=C), 1515 cm⁻¹ (arom.), 1235 cm⁻¹ (C–O–C), 1032 cm⁻¹ (C–O), 987, 950 cm⁻¹ (lactone), 861, 806 cm⁻¹ (C–H), 720 cm⁻¹ (arom.). ^1^H NMR (400 MHz, CDCl_3_) δ 7.85 (s, 1H, −NH), 7.68 (d, *J* = 9.0 Hz, 1H, H‑5), 7.30 (d, *J* = 8.8 Hz, 2H, H‑2′, H‑6′), 6.93 – 6.85 (m, 3H, H‑6, H‑3′, H‑5′), 6.79 (d, *J* = 2.6 Hz, 1H, H‑8), 4.56 (d, *J* = 5.7 Hz, 2H, −CH₂NH−), 3.88 (s, 3H, 7‑OCH₃), 3.79 (s, 3H, 4′‑OCH₃), 2.75 (s, 3H, 4‑CH₃) ppm; ^13^C NMR (101 MHz, CDCl_3_) δ 164.28, 163.83, 160.98, 159.07, 156.78, 154.59, 130.35, 129.18, 127.27, 116.71, 114.21, 114.02, 113.49, 100.44, 56.02, 55.43, 43.42, 16.94 ppm. HR- ESI -MS (m/z):calcd for C_20_H_19_NO_5_ [M + H]^+^: 354.1336, found 354.1341.

**Figure S19**: Infrared spectrum ʋmax (KBr, cm^-1^) of compound **IIb3**

**Figure S20**: ^1^H NMR spectrum (400 MHz, CDCl_3_) of compound **IIb3**

***N*-(3,5-bis(trifluoromethyl)benzyl)-7-methoxy-4-methyl-2-oxo-2*H*-chromene-3-carboxamide (IIb4):** White solid,62% yield; Melting Point: 197.2-200.1°C; **FT-IR (KBr, cm⁻¹):** 3285 cm⁻¹ (N–H), 2849 cm⁻¹ (C–H), 1726 cm⁻¹ (C=O), 1632 cm⁻¹ (C=O), 1385, 1352 cm⁻¹ (CF₃), 1276 cm⁻¹ (C–F), 1188, 1124 cm⁻¹ (C–F), 1034 cm⁻¹ (C–O), 970 cm⁻¹ (lactone), 884, 872, 835 cm⁻¹ (C–H). ^1^H NMR (400 MHz, CDCl_3_) δ 8.47 (s, 1H, −NH), 7.84 (s, 2H, H‑2′, H‑6′), 7.79 – 7.68 (m, 2H, H‑5, H‑4′), 6.93 (d, *J* = 9.0 Hz, 1H, H‑6), 6.80 (s, 1H, H‑8), 4.73 (d, *J* = 6.3 Hz, 2H, −CH₂NH−), 3.88 (s, 3H, 7‑OCH₃), 2.80 (s, 3H, 4‑CH₃) ppm; ^13^C NMR (101 MHz, CDCl_3_) δ 164.21, 163.22, 161.91, 153.54, 152.87, 139.26, 129.40, 129.34, 129.14, 129.08, 127.01, 126.16, 126.12, 121.63, 114.93, 114.49, 110.71, 101.43, 55.66, 44.04, 16.96 ppm. HR- ESI -MS (m/z):calcd for C_21_H_15_F_6_NO_4_ [M + H]^+^: 460.0978, found 460.0983.

**Figure S21**: Infrared spectrum ʋmax (KBr, cm^-1^) of compound **IIb4**

**Figure S22**: ^1^H NMR spectrum (400 MHz, CDCl_3_) of compound **IIb4**

**7-methoxy-4-methyl-2-oxo-*N*-(thiazol-2-yl)-2*H*-chromene-3-carboxamide (IIb5):** Light yellow solid powder,54% yield; Melting Point: 233.9-235.9 °C; **FT-IR (KBr, cm⁻¹):** 3240 cm⁻¹ (N–H), 2940, 2844 cm⁻¹ (C–H), 1709 cm⁻¹ (C=O), 1613 cm⁻¹ (C=N), 1252 cm⁻¹ (C–N), 1020 cm⁻¹ (C–O), 948 cm⁻¹ (lactone), 890, 855 cm⁻¹ (C–H), 728, 685 cm⁻¹ (C–S), 609 cm⁻¹ (C–S). ^1^H NMR (400 MHz, DMSO-*d*_6_) δ 12.54 (s, 1H, −NH), 7.83 (d, *J* = 8.9 Hz, 1H, H‑5), 7.54 (d, *J* = 3.5 Hz, 1H, thiazole‑H‑4′), 7.32 (d, *J* = 3.6 Hz, 1H, thiazole‑H‑5′), 7.09 – 7.01 (m, 2H, H‑6, H‑8), 3.90 (s, 3H, 7‑OCH₃), 2.41 (s, 3H, 4‑CH₃) ppm; ^13^C NMR (101 MHz, DMSO-*d*_6_) δ 163.30, 162.65, 158.28, 157.55, 154.37, 151.17, 138.01, 127.65, 119.02, 114.20, 112.99, 112.35, 100.83, 56.19, 15.95 ppm. HR- ESI -MS (m/z):calcd for C_15_H_12_N_2_O_4_S [M + H]^+^: 317.0591, found 317.0596.

**Figure S23:** Infrared spectrum ʋmax (KBr, cm^-1^) of compound **IIb5**

**Figure S24:** ^1^H NMR spectrum (500 MHz, CDCl_3_) of comp **IIb5**

***N*-(isoxazol-3-yl)-7-methoxy-4-methyl-2-oxo-2*H*-chromene-3-carboxamide (IIb6):** White flake solid,68% yield; Melting Point: 218.8-223.8 °C; **FT-IR (KBr, cm⁻¹):** 3332 cm⁻¹ (N–H), 2944, 2846 cm⁻¹ (C–H), 1685 cm⁻¹ (C=O), 1617 cm⁻¹ (C=N), 1465 cm⁻¹ (arom.), 1264 cm⁻¹ (C–N), 1026 cm⁻¹ (C–O), 917 cm⁻¹ (N–O), 874, 818 cm⁻¹ (C–H), 783 cm⁻¹ (arom.). ^1^H NMR (400 MHz, DMSO-*d*_6_) δ 11.53 (s, 1H, −NH), 8.87 (s, 1H, isoxazole‑H‑5′), 7.82 (d, *J* = 8.8 Hz, 1H, H‑5), 7.10 – 6.96 (m, 3H, H‑6, H‑8, isoxazole‑H‑4′), 3.89 (s, 3H, 7‑OCH₃), 2.42 (s, 3H, 4‑CH₃) ppm; ^13^C NMR (101 MHz, DMSO-*d*_6_) δ 163.12, 162.96, 160.58, 158.21, 157.19, 154.24, 150.33, 127.51, 120.03, 112.87, 112.30, 100.77, 99.18, 56.11, 15.83 ppm. HR- ESI -MS (m/z):calcd for C_15_H_12_N_2_O_5_ [M + H]^+^: 301.0819, found 301.0824.

**Figure S25:** Infrared spectrum ʋmax (KBr, cm^-1^) of compound **IIb6**

**Figure S26:** ^1^H NMR spectrum (500 MHz, CDCl_3_) of comp **IIb6**

**3-(1*H*-imidazole-1-carbonyl)-7-methoxy-4-methyl-2*H*-chromen-2-one (IIb7):** Light yellow solid powder,63% yield; Melting Point: 165.7-171.2 °C; **FT-IR (KBr, cm⁻¹):** 3074 cm⁻¹ (C–H), 2942, 2846 cm⁻¹ (C–H), 1716 cm⁻¹ (C=O), 1609 cm⁻¹ (C=N), 1262 cm⁻¹ (C–O), 1013 cm⁻¹ (C–O), 954 cm⁻¹ (lactone), 907, 868 cm⁻¹ (C–H), 677 cm⁻¹ (arom.).  ^1^H NMR (400 MHz, CDCl_3_) δ 8.03 (s, 1H, imidazole‑H‑2′), 7.67 – 7.61 (m, 1H, H‑5), 7.48 (s, 1H, imidazole‑H‑5′), 7.12 (s, 1H, imidazole‑H‑4′), 6.96 (d, *J* = 8.9 Hz, 1H, H‑6), 6.88 (d, *J* = 1.6 Hz, 1H, H‑8), 3.93 (s, 3H, 7‑OCH₃), 2.44 (br s, *J* = 1.6 Hz, 3H, 4‑CH₃) ppm; ^13^C NMR (101 MHz, CDCl_3_) δ 164.61, 162.04, 158.14, 155.56, 154.02, 137.31, 131.72, 127.01, 117.10, 116.60, 113.87, 112.32, 101.14, 56.09, 16.54 ppm. HR- ESI -MS (m/z):calcd for C_15_H_12_N_2_O_4_ [M + H]^+^: 285.0870, found 285.0875.

**Figure S27:** Infrared spectrum ʋmax (KBr, cm^-1^) of compound **IIb7**

**FigureS28:** ^1^H NMR spectrum (500 MHz, CDCl_3_) of comp **IIb7**

**Chemical characterization of compounds III、IVa-IVd**

**3-amino-7-hydroxy-4H-thieno[3,4-c]chromen-4-one (III):** Purple solid powder,54% yield; Melting Point: 266.7-270.9°C; **FT-IR (KBr, cm⁻¹):** 3400, 3306, 3180 cm⁻¹ (N–H/O–H), 1675 cm⁻¹ (C=O), 1607 cm⁻¹ (C=C), 1502 cm⁻¹ (arom.), 1278, 1159 cm⁻¹ (C–O), 976 cm⁻¹ (lactone), 849, 810, 769 cm⁻¹ (C–H), 666, 621 cm⁻¹ (C–S). ^1^H NMR (400 MHz, DMSO-*d*_6_) δ 9.96 (s, 1H, 7‑OH), 7.71 (s, 2H, −NH₂), 7.66 (d, *J* = 8.5 Hz, 1H, H‑5), 6.66 (dd, *J* = 8.5, 2.4 Hz, 1H, H‑6), 6.59 (s, 1H, thiophene‑H‑2′), 6.57 (d, *J* = 2.4 Hz, 1H, H‑8) ppm; ^13^C NMR (101 MHz, DMSO-*d*_6_) δ 163.31, 158.41, 157.25, 152.74, 137.74, 127.21, 114.17, 112.17, 106.12, 104.28, 101.15 ppm. HR- ESI -MS (m/z):calcd for C_11_H_7_NO_3_S [M + H]^+^: 234.0219, found 234.0227.

**Figure S29:** Infrared spectrum ʋmax (KBr, cm^-1^) of compound **III**

**Figure S30**: ^1^H NMR spectrum (400 MHz, CDCl_3_) of compound **III**

**3-amino-4-oxo-4*H*-thieno[3,4-c]chromen-7-yltrifluoromethanesulfonate (IVa):** Light yellow solid,21% yield; Melting Point: 94.8-96.2 °C; **FT-IR (KBr, cm⁻¹):** 3429, 3300, 3193 cm⁻¹ (NH₂), 1712 cm⁻¹ (C=O), 1609 cm⁻¹ (C=C), 1391 cm⁻¹ (SO₂), 1221 cm⁻¹ (SO₂), 1139, 1118, 1098 cm⁻¹ (C–F), 1057 cm⁻¹ (S–O), 970 cm⁻¹ (skeleton), 880 cm⁻¹ (C–H). ^1^H NMR (400 MHz, CDCl_3_) δ 7.73 (d, *J* = 8.7 Hz, 1H, H‑5), 7.17 (d, *J* = 2.5 Hz, 1H, H‑8), 7.12 (dd, *J* = 8.7, 2.5 Hz, 1H, H‑6), 6.54 (s, 1H, thiophene‑H‑2′), 6.28 (s, 2H, −NH₂) ppm; ^13^C NMR (101 MHz, CDCl_3_) δ 165.24, 159.26, 152.03, 148.99, 130.69, 124.81, 118.52, 117.49, 111.31, 100.53, 98.49 ppm. HR- ESI -MS (m/z):calcd for C_12_H_6_F_3_NO_5_S_2_ [M + H]^+^: 365.9712, found 365.9730.

**Figure S31**: Infrared spectrum ʋmax (KBr, cm^-1^) of compound **IVa**

**Figure S32**: ^1^H NMR spectrum (400 MHz, CDCl_3_) of compound **IVa**

**3-amino-4-oxo-4H-thieno[3,4-c]chromen-7-yl isobutyl carbonate (IVb):** Brown solid,20% yield; Melting Point: 107.5-111.3°C; **FT-IR (KBr, cm⁻¹):** 3458, 3318 cm⁻¹ (NH₂), 2969, 2929, 2881 cm⁻¹ (C–H), 1761 cm⁻¹ (C=O), 1704 cm⁻¹ (C=O), 1225 cm⁻¹ (C–O), 1131 cm⁻¹ (C–O–C), 986 cm⁻¹ (skeleton), 935 cm⁻¹ (skeleton), 888, 767 cm⁻¹ (C–H), 736, 695 cm⁻¹ (heterocycle); ^1^H NMR (400 MHz, CDCl_3_) δ 7.64 (d, *J* = 8.5 Hz, 1H), 7.64 (d, *J* = 8.5 Hz, 1H, H‑5), 7.07 (d, *J* = 2.4 Hz, 1H, H‑8), 7.03 (dd, *J* = 8.5, 2.4 Hz, 1H, H‑6), 6.44 (s, 1H, thiophene‑H‑2′), 6.24 (s, 2H, −NH₂), 4.06 (d, *J* = 6.7 Hz, 2H, −O‑CH₂−), 2.07 (dp, *J* = 13.4, 6.7 Hz, 1H, −CH(CH₃)₂), 1.01 (d, *J* = 6.8 Hz, 6H, −(CH₃)₂) ppm; ^13^C NMR (101 MHz, CDCl_3_) δ 165.14, 159.86, 153.63, 151.90, 151.24, 131.31, 124.07, 117.44, 116.20, 110.81, 100.63, 97.16, 75.28, 27.92, 19.02 ppm. HR- ESI -MS (m/z):calcd for C_16_H_15_NO_5_S [M + H]^+^: 334.0744, found 334.0754.

**Figure S33**: Infrared spectrum ʋmax (KBr, cm^-1^) of compound **IVb**

**Figure S34:** ^1^H NMR spectrum (400 MHz, CDCl_3_) of compound **IVb**

**3-amino-7-((2-methylallyl)oxy)-4*H*-thieno[3,4-c]chromen-4-one (IVc):** Brown solid,25% yield; Melting Point: 113.2-119.7°C; **FT-IR (KBr, cm⁻¹):** 3441, 3349 cm⁻¹ (NH₂), 3098 cm⁻¹ (C–H), 2919 cm⁻¹ (C–H), 1712 cm⁻¹ (C=O), 1615 cm⁻¹ (C=C), 1266 cm⁻¹ (C–O–C), 1161, 1013 cm⁻¹ (C–O), 970, 888 cm⁻¹ (=CH₂), 804, 765 cm⁻¹ (C–H). ^1^H NMR (400 MHz, CDCl_3_) δ 7.62 (d, *J* = 8.5 Hz, 1H, H‑5), 6.90 – 6.79 (m, 2H, H‑6, H‑8), 6.39 (s, 1H, thiophene‑H‑2′), 6.29 (s, 2H, −NH₂), 5.21 – 5.04 (m, 2H, =CH₂), 4.52 (s, 2H, −O‑CH₂−), 1.90 (s, 3H, =C(CH₃)) ppm; ^13^C NMR (101 MHz, CDCl_3_) δ 164.78, 160.51, 159.72, 152.62, 140.41, 132.32, 124.17, 113.36, 112.58, 111.37, 102.90, 101.13, 94.92, 72.17, 19.49 ppm. HR- ESI -MS (m/z):calcd for C_15_H_13_NO_3_S [M + H]^+^: 288.0689, found 288.0713.

**Figure S35:** Infrared spectrum ʋmax (KBr, cm^-1^) of compound **IVc**

**Figure S36:** ^1^H NMR spectrum (500 MHz, CDCl_3_) of comp **IVc**

**3-amino-7-(isopentyloxy)-4H-thieno[3,4-c]chromen-4-one (IVd):** Brown solid powder,40% yield; Melting Point: 115.0-121.1 °C; **FT-IR (KBr, cm⁻¹):** 3437, 3335 cm⁻¹ (NH₂), 2955, 2874 cm⁻¹ (C–H), 1708 cm⁻¹ (C=O), 1621, 1582 cm⁻¹ (C=C), 1266 cm⁻¹ (C–O–C), 1147, 1099, 1055 cm⁻¹ (C–O). ^1^H NMR (400 MHz, CDCl_3_) δ 7.54 (d, *J* = 8.5 Hz, 1H, H‑5), 6.73 (m, 2H, H‑6, H‑8), 6.28 (br m, overlap, 3H, thiophene‑H‑2′, −NH₂), 4.00 (t, *J* = 6.7 Hz, 2H, −O‑CH₂−), 1.83 (hept, *J* = 6.7 Hz, 1H, −CH−), 1.68 (m, 2H, −CH₂−), 0.97 (d, *J* = 6.6 Hz, 6H, −(CH₃)₂) ppm; ^13^C NMR (101 MHz, CDCl_3_) δ 164.84, 160.55, 160.13, 152.64, 132.32, 124.13, 112.34, 111.03, 102.41, 101.04, 94.73, 66.92, 37.90, 25.17, 22.69 ppm. HR- ESI -MS (m/z):calcd for C_16_H_17_NO_3_S [M + H]^+^: 304.1002, found 304.1009.

**Figure S37:** Infrared spectrum ʋmax (KBr, cm^-1^) of compound **IVd**

**Figure S38:** ^1^H NMR spectrum (500 MHz, CDCl_3_) of comp **IVd**

**Supplementary Table**

**Table S1.** Inhibitory rates of coumarin derivatives against four plant pathogenic fungi at 100 μg/mL.

| Compound No | *F. solani* | *L. biglobosa* | *B. cinerea* | *V. mali* |
| --- | --- | --- | --- | --- |
| **IIa1** | 30.68±0.53 | 15.13±1.08 | 23.51±1.98 | — |
| **IIa2** | 7.85±0.82 | 4.26±0.29 | 6.44±0.48 | — |
| **IIa3** | — | 14.05±1.29 | 60.40±2.65 | 15.54±1.58 |
| **IIa4** | — | 2.75±0.24 | 22.69±1.74 | — |
| **IIa5** | 25.69±2.04 | 15.17±1.29 | 58.39±3.20 | 5.43±0.48 |
| **IIa6** | — | 3.21±0.22 | 67.11±1.20 | 12.34±0.86 |
| **IIa7** | 11.72±1.2 | 13.82±0.46 | 65.16±4.62 | 22.51±1.47 |
| **IIb1** | 9.81±0.57 | 5.63±0.36 | 59.15±5.36 | 6.19±0.59 |
| **IIb2** | 7.79±0.68 | 17.62±1.56 | — | — |
| **IIb3** | 17.61±1.5 | 12.75±1.1 | 3.39±0.27 | 6.82±0.67 |
| **IIb4** | 12.90±1.25 | 15.45±0.97 | — | — |
| **IIb5** | 61.91±4.76 | 14.08±1.17 | — | 20.92±1.72 |
| **IIb6** | 7.58±1.52 | 4.47±0.36 | 32.31±2.62 | — |
| **IIb7** | 17.58±2.42 | 10.34±0.82 | 12.35±1.16 | 17.06±1.14 |
| **III** | 25.00±2.80 | 6.84±0.60 | 3.79±0.25 | 14.20±1.32 |
| **IVa** | 31.01±4.63 | 16.67±1.47 | 8.86±0.67 | 36.39±0.88 |
| **IVb** | 11.06±0.84 | 5.13±0.35 | 7.58±0.29 | 16.53±1.25 |
| **IVc** | 14.28±1.26 | 11.97±0.51 | — | 24.88±0.66 |
| **IVd** | 65.45±3.05 | 45.73±2.42 | 19.84±1.62 | 63.56±1.38 |
| **AZ** | 79.26±1.49 | 64.47±5.49 | 84.22±3.40 | 55.47±1.49 |

Antifungal activity was determined by the mycelial growth rate method. Data are presented as mean ± SD (n=3). Abbreviations: *F. solani*, *Fusarium solani*; *L. biglobosa*, *Leptosphaeria biglobosa*; *V. mali*, *Valsa mali*; *B. cinerea*, *Botrytis cinerea;* AZ ,Azoxystrobin.

**Table S2**. EC_50_ values of compound **IVd** and azoxystrobin（AZ） against *V. mali*.

| Compound | Fungal species | Toxicity regression equation | R^2^ | EC_50_  μg/mL |
| --- | --- | --- | --- | --- |
| **IVd** | *V.mali* | Y=0.3362X+20.255 | 0.9268 | 88.5 |
| **AZ** | *V.mali* | Y=0.308X+21.409 | 0.9256 | 92.8 |

**Table** **S3**. Synergistic effects of **IVd** in combination with azoxystrobin (AZ) or fluconazole (FLC) against *V. mali.*

| Group | Toxicity regression equation | R^2^ | EC_50_  （Exp）  μg/mL | EC_50_  （Obs）  μg/mL | SR |
| --- | --- | --- | --- | --- | --- |
| **IVd** | Y=0.3362X+20.255 | 0.9268 | — | 88.47 | — |
| **AZ** | Y=0.308X+21.409 | 0.9256 | — | 92.83 | — |
| **FLC** | Y=0.2413X+32.182 | 0.9756 | — | 73.84 | — |
| **AZ：IVd =** 1:2 | Y=0.2637X+24.141 | 0.9604 | 89.80 | 98.06 | 0.92 |
| **AZ：IVd =** 1:1 | Y=0.1962X+35.008 | 0.9713 | 90.50 | 76.42 | 1.18 |
| **AZ：IVd =** 2:1 | Y=0.2625X+36.043 | 0.9861 | 91.16 | 53.17 | 1.71 |
| **FLC：IVd =** 1:2 | Y=0.2719X+31.86 | 0.9144 | 83.06 | 66.72 | 1.24 |
| **FLC：IVd =** 1:1 | Y=0.2788X+32.301 | 0.9903 | 80.32 | 63.48 | 1.27 |
| **FLC：IVd =** 2:1 | Y=0.3348X+33.381 | 0.9812 | 78.31 | 49.64 | 1.58 |

The results are expressed as the mean values obtained from three independent experiments, with each experiment performed in triplicate. EC₅₀ values were calculated based on dose–response curves using the mycelial growth inhibition data. The R² value represents the coefficient of determination of the toxicity regression equation. The synergistic ratio (SR) was calculated according to the Wadley method. SR > 1.5, synergistic effect; 0.5 ≤ SR ≤ 1.5, additive effect; SR < 0.5, antagonistic effect.

**Table S4**. Binding energies of compound **IVd** with potential target proteins.

| No. | Receptor | PDB code | Affinity (kcal/mol) | Number of H bond |
| --- | --- | --- | --- | --- |
| **1** | CYP51B | 6CR2 | -8.4 | 3 |
| **2** | CYP51 | 5FSA | -8.0 | 2 |
| **3** | SDH | 2FBW | -6.8 | 2 |

Molecular docking was performed using AutoDock Vina. Lower binding energy values indicate higher affinity.
